# Islet sympathetic innervation and islet neuropathology in patients with type 1 diabetes

**DOI:** 10.1038/s41598-021-85659-8

**Published:** 2021-03-22

**Authors:** Martha Campbell-Thompson, Elizabeth A. Butterworth, J. Lucas Boatwright, Malavika A. Nair, Lith H. Nasif, Kamal Nasif, Andy Y. Revell, Alberto Riva, Clayton E. Mathews, Ivan C. Gerling, Desmond A. Schatz, Mark A. Atkinson

**Affiliations:** 1 Department of Pathology, Immunology, and Laboratory Medicine, College of Medicine, University of Florida, Gainesville, FL 32610 USA; 2 Department of Biomedical Engineering, College of Engineering, University of Florida, Gainesville, FL 32610 USA; 3 Bioinformatics Core, Interdisciplinary Center for Biotechnology Research, University of Florida, Gainesville, FL 32610 USA; 4 Department of Medicine-Endocrinology, University of Tennessee Health Science Center, Memphis, TN 38163 USA; 5 Department of Pediatrics, College of Medicine, University of Florida, Gainesville, FL 32610 USA

**Keywords:** Type 1 diabetes, Type 1 diabetes, Autonomic nervous system, Neural circuits

## Abstract

Dysregulation of glucagon secretion in type 1 diabetes (T1D) involves hypersecretion during postprandial states, but insufficient secretion during hypoglycemia. The sympathetic nervous system regulates glucagon secretion. To investigate islet sympathetic innervation in T1D, sympathetic tyrosine hydroxylase (TH) axons were analyzed in control non-diabetic organ donors, non-diabetic islet autoantibody-positive individuals (AAb), and age-matched persons with T1D. Islet TH axon numbers and density were significantly decreased in AAb compared to T1D with no significant differences observed in exocrine TH axon volume or lengths between groups. TH axons were in close approximation to islet α-cells in T1D individuals with long-standing diabetes. Islet RNA-sequencing and qRT-PCR analyses identified significant alterations in noradrenalin degradation, α-adrenergic signaling, cardiac β-adrenergic signaling, catecholamine biosynthesis, and additional neuropathology pathways. The close approximation of TH axons at islet α-cells supports a model for sympathetic efferent neurons directly regulating glucagon secretion. Sympathetic islet innervation and intrinsic adrenergic signaling pathways could be novel targets for improving glucagon secretion in T1D.

## Introduction

In the autoimmune disease, type 1 diabetes (T1D), the progressive loss of functional β-cell mass precipitates clinical onset of hyperglycemia and requires life-long insulin replacement therapy, but dysregulation of glucagon secretion is also present at an early stage and represents a significant impediment to optimal disease management^1–3^. Abnormalities of glucagon secretion manifest as a lack of suppression during hyperglycemic conditions and insufficient secretion during hypoglycemia^4–7^. Importantly, glucagon excess is also present in most patients hospitalized with diabetic ketoacidosis, including those with severe type 2 diabetes (T2D) and hyperglucagonemia may increase the severity of diabetes and thus insulin requirements^8^.

The precise mechanisms underlying dysregulated glucagon secretion in T1D have yet to be defined with specificity. Both the parasympathetic and sympathetic nervous systems are critical regulators of islet hormone secretion to maintain homeostasis and prevent hyperglycemia and hypoglycemia^9–11^. The sympathetic nervous system (SNS) reduces insulin and increases glucagon secretion. However, morphological or functional studies on sympathetic innervation of human α-cells are conflicting^12,13^. Loss of sympathetic innervation was reported in patients with T1D, both at onset and with long-term durations, and was proposed as an explanation for impaired responses to hypoglycemia^14^. In contrast, islet parasympathetic innervation, inferred from pancreatic polypeptide secretion in response to insulin-induced hypoglycemia, appears intact in patients with T1D during the early stages of the disease and correlates with systemic epinephrine levels^15,16^. Direct innervation of α-cells in mouse islets has been reported yet others reported that sympathetic innervation in human islets is solely mediated through effects on the microvasculature rather than α-cells^17,18^. Given the importance of glucagon secretion in managing both hyperglycemia and hypoglycemia in diabetes, the present study was undertaken to characterize the morphology of sympathetic innervation in human pancreatic islets during the progression to T1D and to define islet gene networks associated with adrenergic signaling that may accompany progression to this disease.

## Results

### Human islet sympathetic neuroanatomy in controls

**Figure 1:**
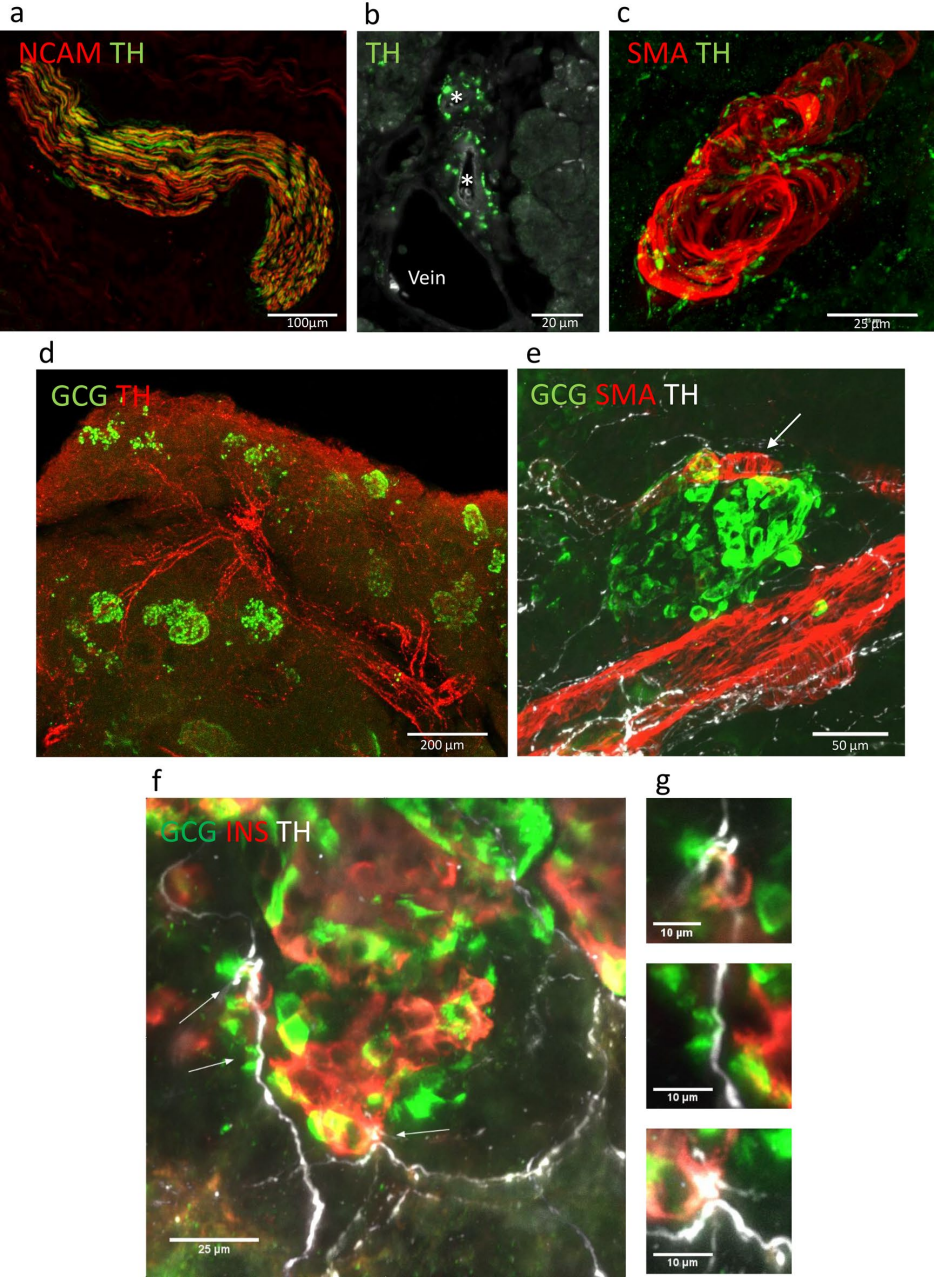
3D human pancreas islet sympathetic neuroanatomy in control donors. (**a**) Maximum project of confocal image for TH axons (red) and NCAM axons (green) in fixed frozen sections showing TH axons with the NCAM bundle. Scale bar 100 µm. (**b**) Two interlobular arteries (asterisks) are shown in cross-section surrounded by numerous TH axons (red). An adjacent vein shows few adjacent TH axons. Scale bar 20 µm. (**c**) Maximum project of confocal image for smooth muscle cells (SMA, red) enveloping an intralobular artery. Surrounding TH axons (green) contain numerous varicosities (see also Supplementary Video 1 for complete z-stack). Scale bar 25 µm. (**d**) Maximum project of confocal image for the pancreatic arterial network outlined with TH (red) axons with branching to islet α-cells (glucagon (GCG), green, iDISCO cleared sample). Scale bar 200 µm. See also Fig. 5. (**e**) Maximum project of confocal image for TH axons and varicosities (white) shown wrapping and coursing from an intralobular artery (SMA, red) and a smaller islet arteriole (white arrow) with adjacent islet α-cells (GCG, green). Scale bar 50 µm. (**f**) Maximum project of confocal image for TH axons and varicosities (white) at a control donor islet stained for insulin (INS, orange) and glucagon (GCG, green). Fibers are observed entering the islet at one pole indicative of the feeding arteriole region with white arrows indicating points of potential contact with endocrine cells. Scale bar 25 µm. (**g**) Points of TH axon contacts with β-cells and α- cells from (**f**) at higher resolutions. Scale bars 10 µm.

**Figure 2:**
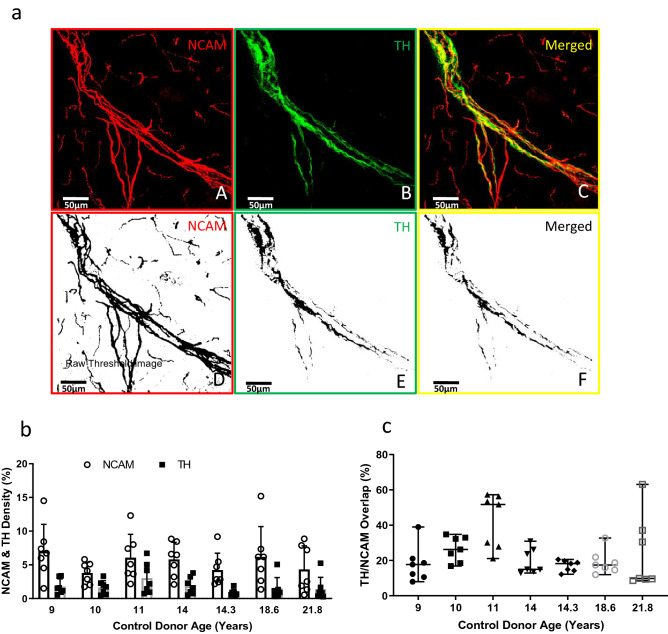
Islet NCAM and TH axon image analysis by confocal microscopy and ImageJ in control donors. Frozen sections (40 µm thick) were fixed and stained for neural cell adhesion marker (NCAM), tyrosine hydroxylase (TH) and glucagon (α-cells, see Fig. 3a) to define total and sympathetic innervation and islets, respectively, as described in Methods. Islets were contoured on maximum intensity projects as demonstrated in (**a**). (**a**) The z-stack confocal images of 40 µm thick sections stained for NCAM and TH were converted into MIP images (A) NCAM in red, (B) TH in green and (C) merged image representing colocalization NCAM and TH. The images D, E, F are the corresponding threshold images to (A), (B) and (C) respectively. These raw threshold images were quantified for % density per unit area for NCAM and TH within islets. (**b**) The thresholded images were quantified for NCAM and TH density (%). (**c**) The thresholded images were quantified for TH/NCAM overlap (%). Data are scatterplots with bars representing mean ± SD for 7 islets/donor in 7 control donors, aged 9–21.8 years old. See also Supplementary Fig. S2.

**Figure 3:**
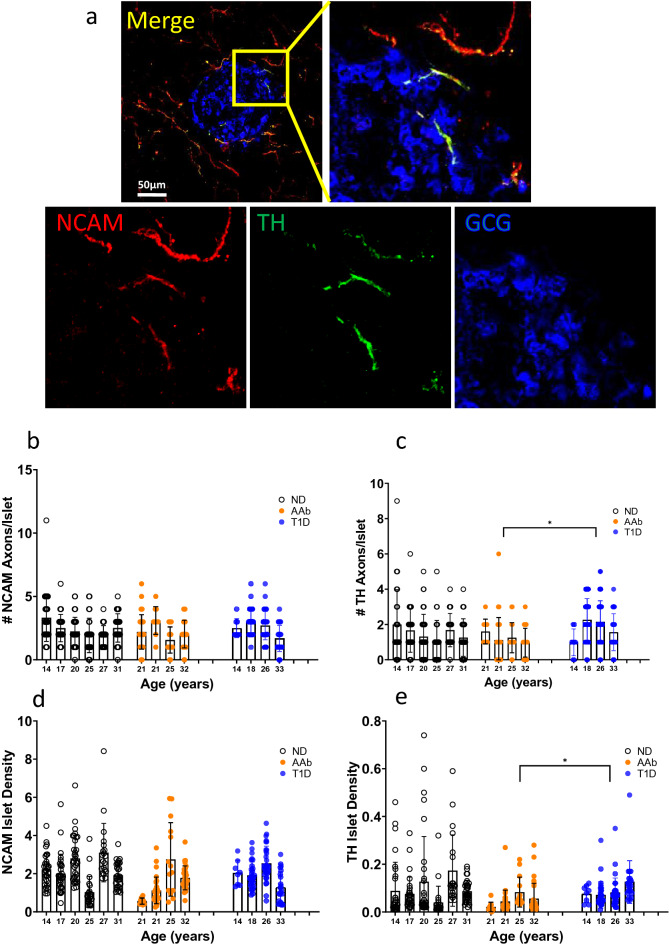
Islet NCAM and TH axon counts and morphometry in T1D. (**a**) Maximum intensity projection of a confocal islet image from a control donor stained by NCAM (red), TH (green), and glucagon (GCG, blue) in a fixed frozen section. A merged image (left upper) with boxed inset for higher detail of intra-islet axons. Single channel images for NCAM and TH show TH axons traveling with NCAM axons into an islet. Scale bar 50 µm. (**b**) NCAM axons per islet were counted (n = 185 islets in 6 ND donors, n = 93 islets in 4 AAb donors, n = 118 islets in 4 T1D donors). (**c**) TH axons per islet were counted (n = 185 islets in 6 ND donors, n = 93 islets in 4 AAb donors, n = 118 islets in 4 T1D donors). *p = 0.05 AAb vs. T1D. (**d**) NCAM axon density within islets was analyzed using ImageJ (n = 167 islets in 6 ND donors, n = 85 islets in 4 AAb donors, n = 93 islets in 4 T1D donors). (**e**) TH axon density within islets was analyzed using ImageJ (n = 167 islets in 6 ND donors, n = 85 islets in 4 AAb donors, n = 93 islets in 4 T1D donors). *p = 0.05 AAb vs. T1D. Data are presented as mean ± SD per donor. All statistical significance values were determined using nested 1-way ANOVA with multiple comparisons. See also Supplementary Fig. S2.

The first objective of these studies was to optimize immunodetection of the islet sympathetic network with high-resolution three-dimension (3D) microscopy and image analysis in control patients. Using both fresh frozen and fixed samples from non-diabetic donors (Supplementary Tables S1, S2), sympathetic axons were delineated by immunostaining for tyrosine hydroxylase (TH), the enzyme responsible for synthesis of norepinephrine (NE) from tyrosine^19,20^. In tandem, we tested several general neural markers including neural cell adhesion molecule 1 (NCAM), UCHL1 (also known as PGP9.5) and TUBB3 (β-tubulin) as markers for total innervation (Supplementary Fig. S1, Supplementary Table S3)^21^. Immunolocalization of NCAM bas been used for the identification of single nerve fibers, nerve bundles, and nerve cell bodies in both the central and peripheral nervous systems^22–24^. As well, NCAM neural structures identified in human colon were reported to be similarly distributed as those staining for UCHL1^25^. NCAM was selected for further use because islet endocrine cells also showed immunopositivity for UCHL1 (Supplementary Fig. S1). Immunostaining with NCAM showed specific labeling from large pancreatic nerve bundles (Fig. 1a) to thin intra-islet axons (Figs. 2a, 3a).

The TH axons traveled with the major pancreatic arteries that branched further into the pancreas parenchyma and continued with wrapping around arterioles to islets (Fig. 1b–e; Supplementary Video 1). The beaded appearance (varicosities) of TH axons indicated high TH concentrations at presumed sympathetic junctions (Fig. 1c,e)^26,27^. The TH varicosities observed at vascular smooth muscle cells were similar to the TH distribution observed adjacent control donor islet β-cells and α-cells (Fig. 1f–g, Supplementary Videos 2, 3).

All TH axons coursed within NCAM nerve bundles (Fig. 2a, Supplementary Fig. S2). Quantification of NCAM and TH axons in islets from the pancreas tail region was performed in control donors aged from 9.0 to 21.8 years old using z-stacks of confocal images. All islets had ≥ 1 NCAM axons and numbers of NCAM axons/islet were 2.57 ± 1.46 (mean ± SD, n = 7 islets per donor, 7 donors). Numbers of TH axons/islet were 1.71 ± 1.46. Density of NCAM and TH axon area per islet area showed similar averages with variability within and between donors (Fig. 2b). The overlap (%) of TH axons with NCAM fibers varied (23.82 ± 13.67, Fig. 2c). These data show that TH axons comprise ~ 25% of the NCAM islet innervation in control subjects and likely innervation of both islet vasculature and endocrine cells. We did not observe human islet endocrine cells with TH immunoreactivity as reported for multiple other species^17,28–31^.

### Altered islet sympathetic neuroanatomy in AAb and T1D individuals

Islet NCAM and TH axon numbers, densities, and morphometry were next determined in fresh frozen samples from donors in the three study groups (Fig. 3a, Supplementary Tables 1, 2). NCAM and TH axon numbers per islet were determine in each group [n = 185 islets in 6 non-diabetic (ND) donors, n = 93 islets in 4 autoantibody-positive (AAb) donors, n = 118 islets in 4 T1D donors]. Numbers of NCAM axons per islet were similar between groups (Fig. 3b) however numbers of TH axons per islet were significantly fewer in AAb donors compared to T1D individuals (Fig. 3c). Specifically, numbers of NCAM axons/islet were 2.44 ± 1.36 in ND, 2.13 ± 1.24 in AAb, and 2.49 ± 1.17 in T1D (n = 396 islets) and numbers of TH axons/islet were 1.49 ± 1.37 in ND, 1.14 ± 1.02 in AAb, and 1.94 ± 1.19 in T1D donors.

NCAM and TH axon density within islets was analyzed using ImageJ (n = 167 islets in 6 ND donors, n = 85 islets in 4 AAb donors, n = 93 islets in 4 T1D donors). Variable differences were observed for axon densities as for axon numbers. Islet NCAM axon densities were similar between groups (Fig. 3d) while TH axon density was significantly lower in AAb donors compared to T1D individuals (Fig. 3e).

The morphometry of islet NCAM and TH axons were further analyzed using ImageJ (n = 10 islets per donor, N = 4 ND donors; n = 10 islets/donor, N = 4 AAb donors; n = 8–10 islets/donor, N = 4 T1D donors). NCAM and TH axon diameters and lengths were similar between all 3 groups (Supplementary Fig. S3). The tortuosity of islet TH axons was examined by determination of axon branch numbers and they were also not significantly different between groups (Supplementary Fig. S3). Thus, TH axons were in lower numbers and density in AAb islets in comparison to T1D islets but without significant differences compared to controls. Islet NCAM and TH axons were also similar in diameters, lengths and number of branches between groups. Further studies are indicated to examine the potential for TH axonal remodeling in AAb islets. Importantly, these data do not confirm a previous report of loss of TH innervation in patients with long-standing T1D as these donors did not have insulin-positive islets by histopathology (Supplementary Table 2)^14^.

### Islet sympathetic axons varicosities are proximal to α-cells and δ-cells in T1D

Our hypothesis for dysregulated glucagon secretion in individuals with T1D is that alterations exist in sympathetic input to islet α-cells. To examine sympathetic input to islet endocrine cells, TH axons and their varicosities were assessed by confocal microscopy for proximity to islet endocrine cells. Islet endocrine cells express two synaptic markers, synaptophysin (SYP) and synapsin 1 (SYN), and their expression patterns were examined for use as a marker for putative sympathetic synaptic junctions (Supplementary Fig. S1). In a human sympathetic celiac ganglia, SYP immunopositivity was found in varicosities immediately adjacent to TH cell bodies and axon. SYP immunopositivity was also highly expressed in all human and mouse islet endocrine cells, as was SYN, thus neither could not be used to define individual sympathetic synapses at islet endocrine cells. Further details for species differences related to islet innervation can be found in several excellent reviews^32–35^.

**Figure 4:**
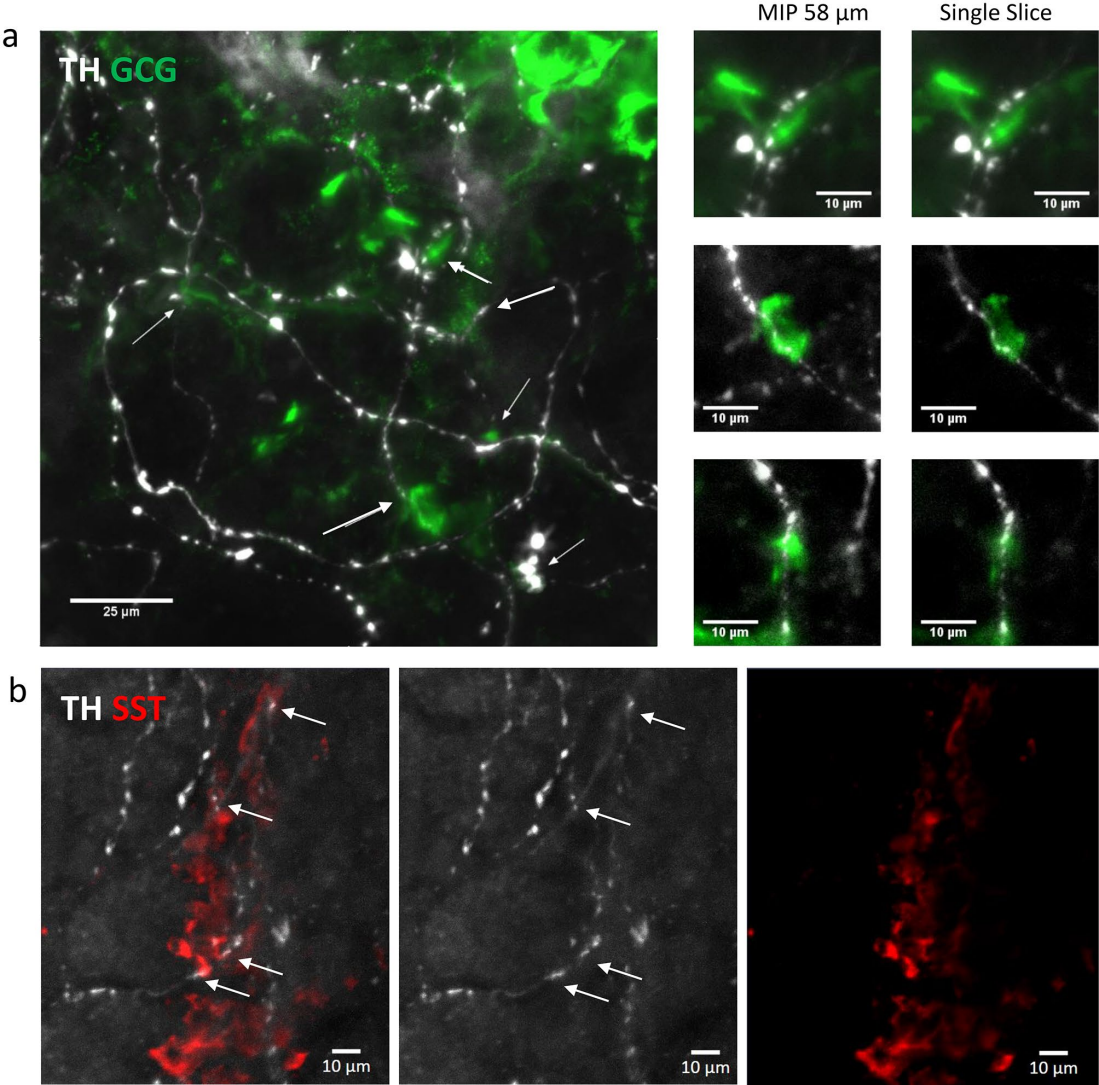
Islet α-cell and δ-cell proximity to TH varicosities in T1D. (**a**) Maximum project for human islet stained for TH (white) and GCG (green) in a T1D individual. TH axons in close proximity to α-cells are indicated by white arrows. Scale bar 25 µm. Insets for maximum intensity project (MIP, 58 µm stack) and single z slices for three regions in (**a**) are shown in higher detail. Scale bars 10 µm. (**b**) Maximum project for human islet stained for TH (white) and somatostatin (SST, red) in a T1D individual. A TH fiber coursing in close proximity to δ-cells (white arrows) is shown by merged and single channels. Scale bar 10 µm. See also Supplementary Videos 2–3.

Using confocal microscopy, the TH varicosities were in close proximity to multiple islet α-cells in islets from patients with long standing T1D (Fig. 4a). Islet somatostatin δ-cells in T1D patients showed similar TH varicosities in close proximity (Fig. 4b). Our data are thus supportive for a direct role of sympathetic neurons on islet endocrine cell function via neurotransmitter diffusion from adjacent TH synaptic junctions. While morphologically intact islet TH axons were observed in islets without β-cells in these T1D individuals, their medical history related to glucagon secretion or hypoglycemia episodes is not known or available.

### Sympathetic neuroanatomy in the exocrine pancreas

**Figure 5:**
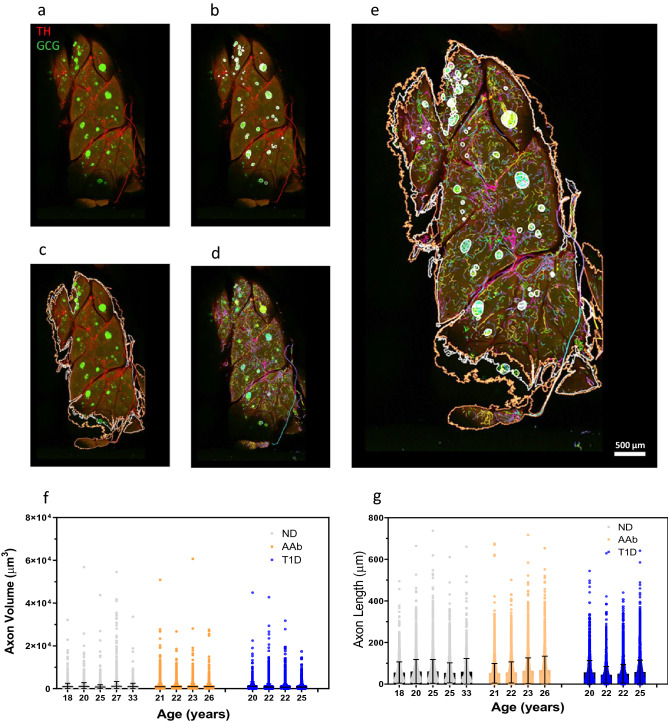
Exocrine pancreas iDISCO image analysis pipeline and TH axon volume and length. (**a**) Maximum intensity projection of a control pancreas slice is shown depicting key image analysis steps with Neurolucida360. TH axons (red) and α-cells (GCG, green) delineate sympathetic axons and islets, respectively. (**b**) Islets are contoured (white) based on GCG signal. (**c**) Slice outlines are shown contoured in white and orange for the first and last slices, respectively, and all slice outlines were used to determine total sample volume (µm^3^). (**d**) TH axons were automatically traced following thresholding and individual axons were displayed in separate colors. (**e**) Merged image showing overlays of TH axons and islet and slice contours. Scale bar: 500 µm. (**f**) Exocrine TH axon volumes (µm^3^) were not significantly different between groups. (**g**) Exocrine TH axon lengths (µm) volumes were not significantly different between groups. Data were plotted for all TH axon volumes and lengths per donor (n = 1381–8197 axons/donor) for 4 donors in each group. Statistical significance was tested using 1-way ANOVA for nested tables.

We used optical clearing (iDISCO) methods and large tissues slices and 3D image analysis by Neurolucida360 to test for alterations in exocrine TH axon volume and length between the three groups (4947 ± 2348 axons/donor (range 1381–8197/donor), n = 5 ND, n = 4 AAb, n = 4 T1D). The workflow for the image analysis is shown in Fig. 5a–e. The range of TH axon volumes was highly variable as expected due to the detection of large TH fiber tracts traveling with major arteries and branching to progressively thinner terminal axons (Fig. 1d). Significant differences in exocrine TH volumes were not detected between groups (Fig. 5f). The TH axon lengths were similarly variable and significant differences were also not observed between groups (Fig. 5g). In sum, significant differences in exocrine TH axon morphometry were not observed between groups.

### Differential gene expression by RNA-seq supports islet neuropathology in T1D

**Figure 6:**
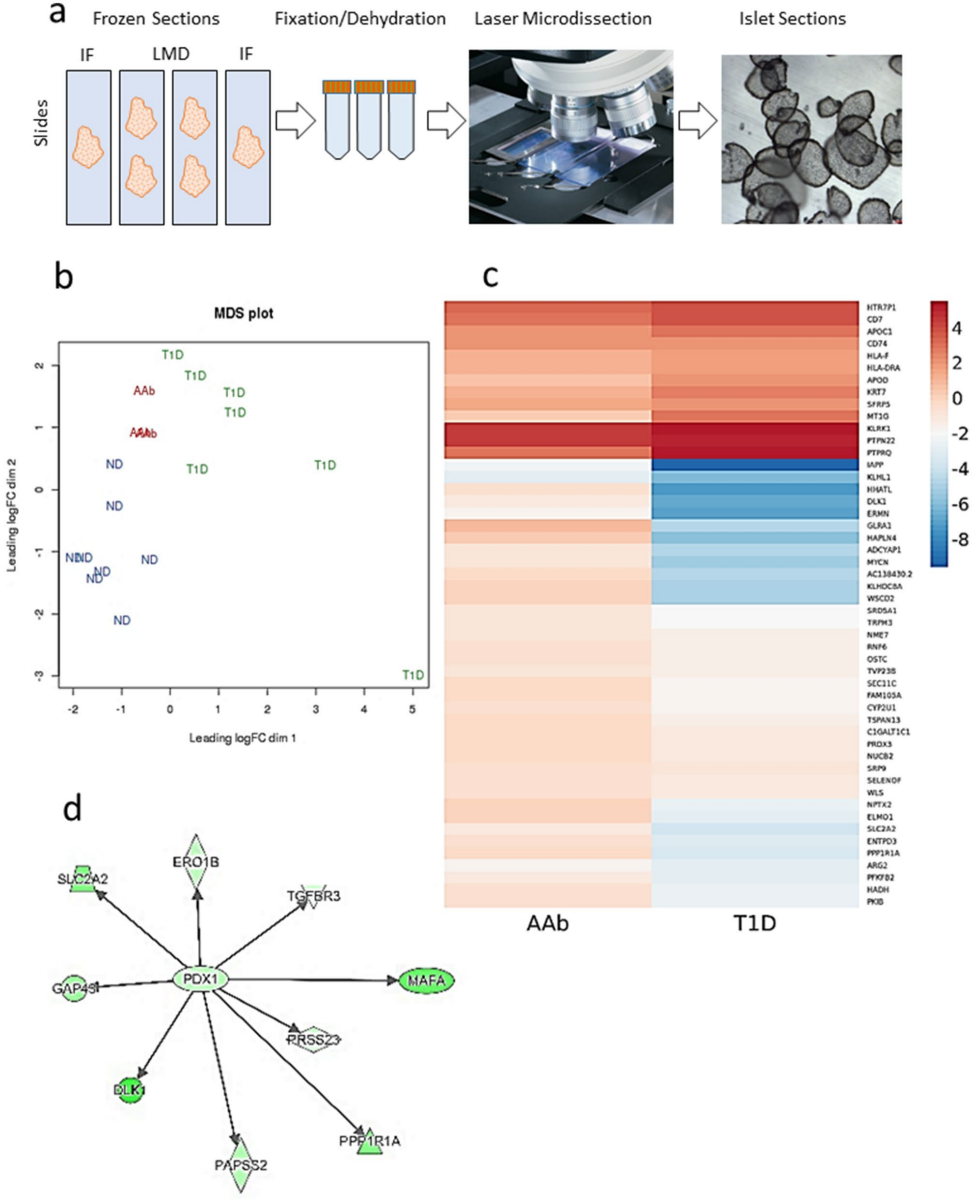
Laser microdissection assay pipeline and RNAseq analysis. (**a**) Experimental RNA-seq pipeline. Fresh frozen pancreas sections were placed onto slides and following fixation and dehydration, islets were microdissected using a Leica LMD7000 microscope. Microdissected islets were visualized in the microvial cap (far right). (**b**) Multi-dimensional scaling (MDS) plot shows grouping of donors within groups (n = 8 ND, n = 3 AAb, n = 7 T1D). One T1D donor (lower right) was not excluded though appearing as an outlier due to additional QC (see Supplementary Fig. S4). (**c**) Heatmap of top 50 differentially expressed genes between AAb versus ND (AAb) and T1D versus ND (T1D) (blue, down-regulated; red, upregulated). (**d**) Network interaction plot shows the transcription factor *PDX-1* with other gene interactions including *SLC2A2* (*GLUT2*), *MAFA* and *TGFBR3* (green, down regulated; red, upregulated). See Supplementary Tables 4–5 for gene lists.

Complexities of paracrine signaling between islet endocrine and accessory cells, as well as inputs from both parasympathetic and SNS limit understanding of the mechanisms involved in neural regulation of and neurotransmission in human α-cells. While single-cell studies are revolutionizing understanding of islet cellular heterogeneity, we reasoned that laser microdissection of in situ human islets (Fig. 6a) followed by islet RNA-seq could provide an important initial step to better understand SNS-related gene pathways as we would avoid the inherent stress of islet and single-cell isolation procedures^36^. All islets used in this study from ND and AAb donors contained β-cells while only rare islets from the T1D donors had a few residual INS-positive β-cells; thus, we also used this model to better understand how loss of paracrine insulin secretion could impact α-cell gene expression. Quality of the RNA-seq datasets was confirmed using a multi-dimensional scaling (MDS) plot showing very good grouping of donors by type, except for one T1D donor (n = 8 ND, n = 3 AAb, n = 7 T1D) (Fig. 6b); we elected not to exclude this donor based on our additional QC parameters which demonstrated that expression values were highly consistent across the range of expression for all samples (Supplementary Fig. S4). Consistent with the MDS plot (Fig. 6b), which indicated that AAb gene expression values fell largely intermediate to both ND and T1D, pairwise *t*-tests revealed few differences in differentially expressed (DE) genes between AAb versus ND or AAb versus T1D (Supplementary Fig. S4). Conversely, the T1D versus ND contrast exhibited 89 DE genes (Supplementary Fig. S4).

RNA-seq with DE gene and gene set enrichment analysis (GSEA) identified significant alterations in canonical neural pathways between T1D and ND donors that included noradrenalin and adrenalin degradation, α-adrenergic signaling, cardiac β-adrenergic signaling, catecholamine biosynthesis, as well as alterations in several neurotransmitter pathways (serotonin, GABA, dopamine, nNOS; Supplementary Table S4). Top DE genes are provided for the three pairwise comparisons (Supplementary Table S5). A hierarchal map for the top 50 DE genes across all donor samples is shown for AAb-ND and T1D-ND comparisons (Fig. 6c). When DE genes were grouped by diseases and functions, nine of the top ten ranked categories were associated with neurological disease in the T1D-ND comparison. Neuromuscular disease was the most significantly dysregulated category with 101 molecules (p = 2.39E−15). Diabetes mellitus was the eighth-ranked category with 77 molecules (p = 3.33E−08). Upstream regulator analyses showed *PDX1* as the top DE transcription factor (p = 9.71E−07, Log ratio − 1.345). Interactions of *PDX1* with target genes included *SLC2A2* (GLUT2), *MAFA* (β-cell specific), and *TGFBR3* (Fig. 6d). The second DE upstream regulator was *IFNA* (p = 1.39E−04). Network analyses showed numerous DE molecules associated with cell development, endocrine system development and function, cell-to-cell signaling and interaction (T1D-ND, AAb-ND), immunological and neurological diseases (AAb-T1D) as well as nervous system development and function (AAb-ND).

Three DE genes between AAb versus T1D donors included *MT1G* (metallothionein 1G, down-regulated), *GLRA1* (glycine receptor alpha 1, upregulated), and *ELMO1* (engulfment and cell motility 1, upregulated) (Supplementary Table S5). Metallothioneins bind various metals including zinc, which has been proposed as a negative intra-islet regulator of glucagon secretion in addition to having key roles in insulin biosynthesis and storage^37^. Interestingly, *MT1G* expression was significantly upregulated in islets from T2D subjects obtained by laser capture microdissection^38^. *ELMO1* is required for phagocytosis of apoptotic cells and cell motility, and single nucleotide polymorphisms (SNPs) tagged to *ELMO1* are associated with diabetic nephropathy in T1D and T2D^39,40^. Upregulation of *ELMO1* expression is associated with increased cell stress; thus, its downregulation in AAb islets may represent enhanced cell protection responses. *GLRA1* encodes a glycine receptor that acts as a ligand-gated ion channel, and loss of *GLRA1* impairs glucose stimulated insulin secretion^41^. All three of these genes were regulated by the E2F family of transcription factors including *E2F1* and *E2F2*. Interestingly, *E2f1*/*E2f2* double knockout mice develop diabetes as well as exocrine atrophy^42,43^, and decreased acinar cell numbers were reported in human T1D in reference to loss of pancreas size.

Of the six DE genes between AAb and ND donors, the most interesting one in terms of autoimmune diseases is *CD74*, also known as HLA Class II antigen associated invariant chain, which is part of the class II major histocompatibility complex (MHC). RNA-seq showed significant upregulation of CD74 in both AAb and T1D donors as compared to ND donors (Supplementary Table S5), in agreement with a recent study that reported increased expression of CD74 in β-cells from T1D donors^44^. Our study is the first report of increased CD74 gene expression in AAb donors.

### Targeted gene expression analysis corroborates neuropathology in AAb and T1D islets

**Figure 7:**
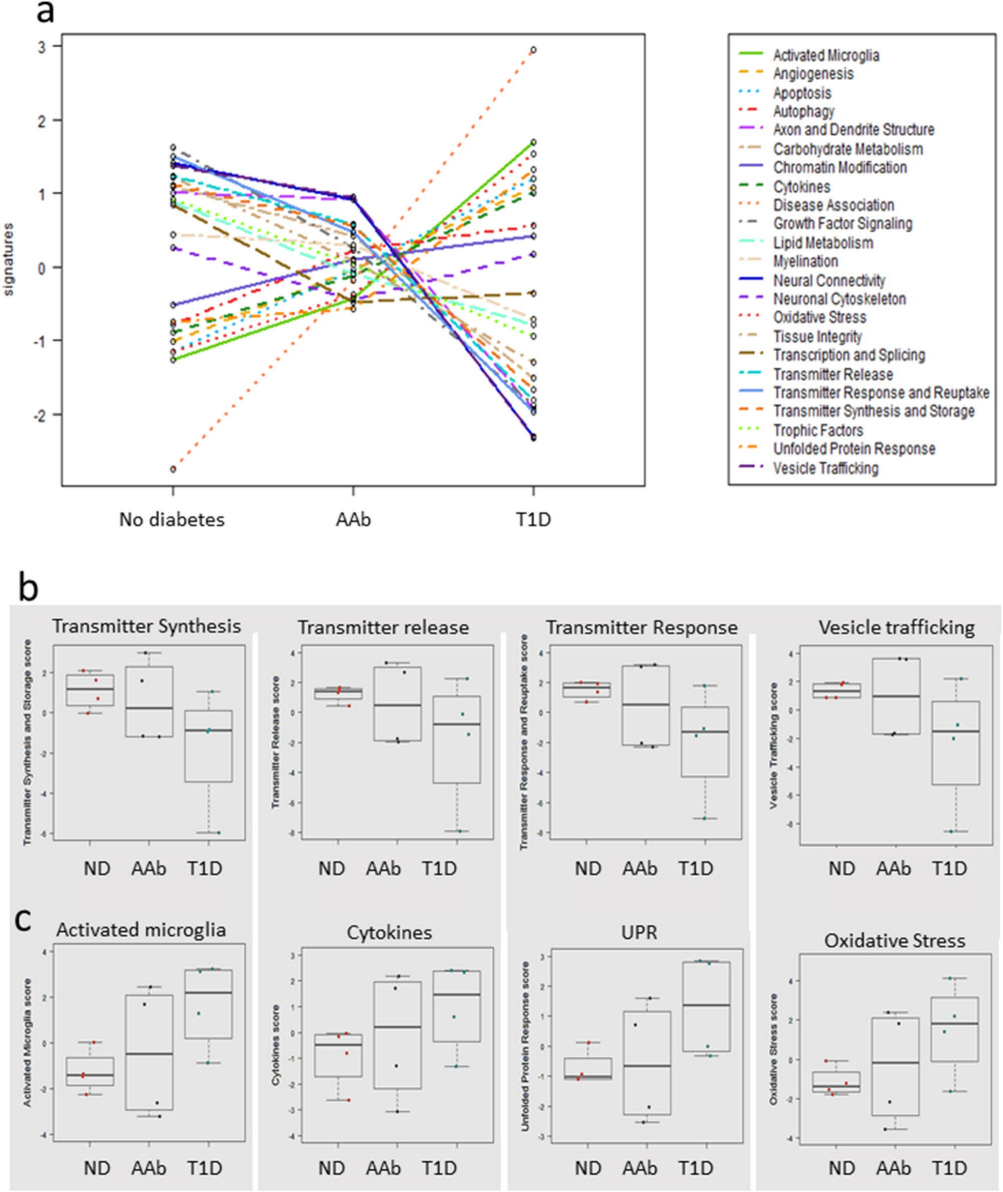
Islet neuropathology pathways in AAb and T1D donors. (**a**) Nanostring neuropathology pathways with significantly different gene regulation between the donor groups are shown by colored line plots with pathways listed in the side-box (n = 4 no diabetes, n = 4 Autoab Pos (AAb), n = 4 T1D donors). (**b**) Bar and whisker plots (median, range) of relative gene expression levels for transmitter synthesis and storage, transmitter release, transmitter response and update, and vesicle trafficking that were significantly downregulated in T1D donors. Donor types are ordered as in (**a**) (**c**) Bar and whisker plots (median, range) of relative gene expression levels for activated microglia, cytokines, unfolded protein response (UPR) and oxidative stress pathways that were significantly upregulated in T1D donors. See Supplementary Tables S6–7 for additional details.

Targeted Nanostring nCounter analysis of neurological gene pathways expressed in microdissected islets was performed from four donors in each of the three groups. Six fundamental categories are represented in the Nanostring panel including genes representing neurodegeneration, neurotransmission, neuron-glia interaction, neuroplasticity, cell structure integrity, neuroinflammation, and metabolism. The neuropathology gene panel contains 770 genes including the β-cell specific genes INS and GAD2, TH, numerous markers for neurotransmitters, neurotransmitter receptors and transporters, immune cells, interleukins and receptors, and growth receptors. QC metrics showed very good negative control (cut-off < 21) and housekeeping gene counts (Supplementary Fig. S5, cut-off > 100 for 8 of 10 genes). Key pathways showing significant down regulation included transmitter synthesis and storage, carbohydrate metabolism, unfolded protein response, and transcription and splicing. Differential gene expression showing significant up-regulation included pathways associated with autophagy, activated microglia, tissue integrity, and cytokines. Interestingly, pathways showing differentially regulation between AAb and T1D donors for axon and dendrite structure, oxidative stress, neural connectivity, as well as neurotransmitter synthesis and response (Fig. 7a, Supplementary Tables S6–7), suggesting progressive worsening of islet neuropathology in the progression to T1D development. In T1D and AAb versus ND islets, significant down regulation was observed for pathways associated with neurotransmitter synthesis and storage, transmitter release, transmitter response and reuptake, and vesicle trafficking (Fig. 7b). Pathways associated with activated microglia and unfolded protein response (UPR) were also significantly dysregulated in T1D and AAb samples versus ND (Fig. 7c). In summary, islet endocrine cells in the natural history of type 1 diabetes exhibited DE patterns associated with mechanisms of neurodegeneration.

## Discussion

Glucagon is released from islet α-cells in response to hypoglycemia, amino acids, catecholamines (NE, epinephrine), other hormones, and neurotransmitters from both the parasympathetic and sympathetic branches of the autonomic nervous system^9,10^. Increased sympathetic tone contributes to glucose counterregulatory responses to hypoglycemia by stimulating islet glucagon secretion and inhibiting insulin secretion^10^ while parasympathetic mechanisms mediate the cephalic phase of islet hormone section that are critical in anticipatory and postprandial blood glucose control^45^. Hypoglycemia is detected within the central nervous system, and counterregulatory responses are mediated by the SNS that stimulate release of NE by the adrenal cortex into the systemic circulation and by other sympathetic neurons including those of the celiac ganglia. The post-ganglionic sympathetic axons travel as splanchnic nerves from the celiac ganglia and enter the pancreas with its major arteries as neurovascular bundles^46^. Sympathetic neuron activation mediates multiple effects in the pancreas including vasoconstriction, reduced insulin secretion and increased glucagon secretion^6,7^.

The importance of glucagon secretion in the etiology of T1D was first highlighted in 1975 where investigators hypothesized that T1D results both from loss of β-cell mass and from excessive glucagon secretion^47^. Several subsequent studies demonstrated postprandial hyperglucagonemia is problematic in T1D patients as well as those with T2D^48,49^. An intrinsic α-cell defect was first suggested by Gerich, et al. who reported no increase in glucagon secretion during severe insulin-induced hypoglycemia in six juvenile patients with T1D, yet excessive glucagon responses with arginine infusion^50^. Recent studies report that in adults with T1D, α-cell responses to hypoglycemia are only observed in patients with high levels of residual C-peptide production^51^. In vitro studies utilizing islets isolated from patients with T1D confirm an intrinsic α-cell secretory defect^52,53^.

Two main hypotheses proposed for loss of appropriate glucagon secretion during hypoglycemia include loss of sympathetic innervation to stimulate islet glucagon secretion and intrinsic defects in α-cells, either primary or secondary to loss of islet β-cells. To our knowledge, the data presented herein provide the first quantification of sympathetic TH axons in human islets during the progression to T1D. Islet TH axon numbers were significantly lower in AAb donors compared to T1D individuals, while exocrine TH axon density was unaltered. We also provide addition evidence for α-cells and δ-cells being in sufficiently close proximity to TH axons to support a hypothesis for direct sympathetic stimulation of these islet endocrine cell types in addition to β-cells and non-endocrine islet cell types. Others reported no direct contact between sympathetic nerves and islet endocrine cells in humans and proposed that modulation of islet blood flow was the only effect mediated by sympathetic stimulation^18^. It remains unclear how sympathetic-mediated decreased islet blood flow would stimulate α-cell glucagon secretion in individuals thus further studies are clearly needed. Decreased blood flow in the exocrine pancreas would be expected to parallel decreased exocrine secretions observed with pancreatic sympathetic stimulation^54^.

We also report decreased sympathetic axon density in islets from AAb + donors compared to T1D patients. These findings may assist with understanding how glucagon responses can be markedly impaired at onset of T1D or alternatively, reflect changes in innervation secondary to the autoimmune attack underlying loss of β-cell mass that occurs during the months and years leading up to clinical onset^55^. Following T1D onset, neural remodeling may occur and thus partly explain the increase in numbers and density of TH axons in T1D islets compared to AAb donors. In one study reporting a marked loss of sympathetic nerves to islets in patients with T1D, the authors proposed that a loss in sympathetic innervation resulted in the loss of glucagon secretion in response to hypoglycemia (Table 1)^56^. In that study, samples were collected from autopsies of young patients with recent-onset T1D during the 1960s in Scotland, and studies were performed on thin paraffin sections. Thus, some major technical differences exist between the two studies both in terms of sample sources (autopsy versus organ donor) and sample type (thin paraffin section versus thick fresh frozen and fixed pancreas sections). Conflicting results are also present for studies in rodents and canines and are summarized in Table 1.

**Table 1:** Sympathetic innervation of islets in T1D.

Islet sympathetic nerve changes	Species (model)	First author	Year	Journal	Major similarities	Major differences	References
Fewer contacts	Rat (STZ, Biobreeder)	Tominaga, M	1987	Diabetes	Electron microscopy (EM): Intact sympathetic nerve endings	EM: Fewer α-cell contacts	^57^
Loss	Rat (Biobreeder)	Mei, Q	2002	Diabetes	Cryosections (16 µm)	Primary antibody: VMAT2	^58^
Loss	Mouse (NOD)	Persson-Sjögren, S	2005	J Neuroimmunol	Cryosections (8 µm)	Primary antibody: UCHL1; islet acetylcholinesterase activity	^59^
Loss	Mouse (NOD)	Taborsky, G.J	2009	Diabetologia	Cryosections (16 µm)	Primary antibody: NPY	^60^
Loss	Human	Mundinger, T.O	2016	Diabetes	Primary antibody (TH, Millipore)	Autopsy specimens; Samples: 4 µm formalin-fixed paraffin	^14^
Loss	Canine (spontaneous diabetes)	Gilor, C	2020	Scientific Reports		Necropsy samples; Primary antibody: Rat anti-TH (PelFreeze Biologicals); Samples: 4 µm formalin-fixed paraffin	^61^
Increased, remodeling	Mouse (STZ, NOD)	Chiu, Y.-C	2012	Diabetologia	Primary antibody (TH, Millipore); Optical clearing 400 µm sections		^17^
Increased	Mouse (NOD)	Alvarsson, A	2020	Science Advances	Primary antibody (TH, Millipore); Optical clearing mm thick sections		^62^

Studies in animal models and human demonstrating alterations (loss or no loss/increase) in islet innervation in T1D.

To aid in understanding potential intrinsic mechanisms that may be responsible for abnormal α-cell responses, gene expression studies were also performed. Multiple lines of evidence show that dysfunctional α-cells contribute to the pathogenesis of diabetes, yet the emphasis on loss or dysfunction of β-cells remains central to most studies^2^. Hypersecretion of glucagon has been explained by the loss of a paracrine signal from adjacent β-cells because insulin secretion normally inversely regulates glucagon secretion^10,63^. Proposed candidates include insulin itself, zinc associated with insulin molecules, other β-cell secretory products such as gamma-aminobutyric acid (GABA), or direct contact between islet β- and α-cells^64^. In terms of alterations in α-cell gene expression, a previous study found downregulation of multiple genes important for α-cell identity and functional deficits with implications response to hypoglycemia^52^. Our studies identified DE genes and pathways associated with sympathetic neurotransmission. Sympathetic innervation of islets reciprocally regulates hormone release in response to hypoglycemia by inhibition of insulin release through activation of α2-adrenergic receptors (ADRA2A) on β-cells and stimulation of glucagon secretion by activation of β2-adrenergic receptors (ADRB2) on α-cells^65^. Another study showed that β1 adrenergic receptor (ADRB1*)* mRNA was found exclusively in α-cells, while ADRB2 were expressed in α-cells as well as a number of other endocrine islet cells^66^. Our RNA-seq and Nanostring data showed expression of adrenergic receptors was decreased in microdissected T1D islets however, their expression levels were near the minimum cut-off levels for both assays. Our RNA-seq data indicate the importance of being able to differentiate expression noise from true, small-effect differences in gene expression between groups since there are clearly differences between the pairwise AAb contrasts. Due to limitations in either sample or effect size, we were unable to finely resolve DE genes between AAb and the other two donor groups.

Whole islet RNA-seq does not provide single cell resolution, and single cell RNA-seq studies are expanding understanding of high islet single cell heterogeneity^67–69^. The majority of islets isolated by laser capture microdissection for the T1D donors used in this study were devoid of β-cells determined by immunohistochemistry staining (Supplementary Table S2), yet we did not completely exclude β-cells as indicated by the presence of low amounts of insulin mRNA detected by both RNA-seq and NanoString. Because low sample numbers from AAb individuals were used in the transcriptomic studies, namely three for RNA-seq and four for qRT-PCR assays, these data are considered preliminary yet nevertheless represent the first published datasets on islet RNA-seq for AAb individuals.

Understanding the SNS regulation of human α-cells has important implications for pathogenic mechanisms underlying dysregulation of glucagon secretion in patients with T1D related to hyperglycemia and hypoglycemia. A limitation of the present study is that glucagon levels in these organ donors are unknown and thus the functional status of sympathetic regulation of islet α-cells secretion remains untested. Importantly, our work does suggest an intact islet sympathetic efferent innervation along with intrinsic alterations in islet genes related to adrenergic signaling after onset of T1D. Further studies are needed to better understand these mechanisms, their relevance and implications of the SNS on human islet α-cell biology as well as the exocrine pancreas during the progression to T1D.

## Materials and methods

### Subjects

Human pancreas samples were recovered from cadaveric organ donors by Organ Procurement Organizations partnering with the Network of Pancreas Organ donors with Diabetes (nPOD) program, University of Florida (UF), in accordance with research donation laws and regulations. The study protocol was classified as “Non-Human Subjects” by the UF Institutional Review Board (IRB) (IRB 392-2008), waiving the need for consent^70^. The nPOD samples used in this specific study were approved as nonhuman by the UF IRB (IRB201902530).

The pancreas was recovered as if for clinical transplantation and shipped in cold transport media for ≤ 24 h before processing. Clinical diagnosis for donor type, with respect to diabetes, was according to American Diabetes Association criteria with information obtained for laboratory testing for serum c-peptide levels and autoantibodies (Supplementary Table S1)^70^. Samples were selected from three types of donors: (1) autoantibody-negative controls without pancreatic disease or diabetes history (non-diabetic, ND), (2) autoantibody-positive donors without diabetes (AAb), and (3) donors with T1D (Supplementary Table S1). The AAb and T1D donors were matched with ND donors for age, gender, and ethnicity as much as feasible. Pancreas fractional insulin and glucagon areas (from Ref.^71^) and histopathology results are listed including the detection of insulin-positive (INS+) islets or insulitis and overall islet histological findings (Supplementary Table S2).

### Mouse pancreas

All procedures were accomplished in agreement with the published guidelines and regulations of the National Institutes of Health for the care and use of laboratory animals. The protocol was approved by the UF Institutional Animal Care and Use Committee (IACUC 202009976).

Formalin fixed paraffin embedded (4 µm) and fresh frozen samples (40 µm) from a C57B6/J mice were used for validating some primary antibodies.

### Human pancreas samples and sectioning

Samples from the distal tail region were used to minimize inter-donor variability for regional differences in islet density and innervation previously reported for human pancreas^72,73^. Fresh frozen sections (40 μm) were prepared from fresh frozen blocks and stored at − 80 °C until use. Trimmed pancreas samples (~ 1–2 cm^3^) were fixed in cold 4% paraformaldehyde (PFA) in PBS for 48 h followed by several washes with PBS. Pancreas slices were manually (1–2 mm) or vibratome sectioned (500 μm) following 4% agarose embedding using a VT100S vibratome (Leica). Slices were stored in 0.1% PFA/PBS at room temperature until use.

### Optical clearing

Optical clearing was performed using two methods: (1) manually cut sections were embedded in acrylamide hydrogel and passively cleared as previously described (PACT)^74^ or (2) vibratome sections were optically cleared by a modified iDISCO procedure (HSK-1, Visikol Inc.) (https://dx.doi.org/10.17504/protocols.io.ba4qigvw). PACT cleared samples were used primarily for initial studies testing antibody specificity and iDISCO was used for exocrine TH axon morphometry.

### Immunostaining

Samples for the initial study for control donors was performed in batches of 1–2 slides per assay while the slides and pancreas slices used for studied of islet and exocrine innervation, respectfully, were stained in batch assay. Samples were stained using multiplex immunofluorescence for endocrine cells [insulin (INS), glucagon (GCG), somatostatin (SST), secretogranin 3 (SCG3)], vasculature [smooth muscle actin (SMA)], neurons and their axons (NCAM, TH) and synapses [synaptophysin (SYP), synapsin 1 (SYN)] (Supplementary Table S3). The optical cleared samples were washed multiple times PBST before staining as previously reported^74^. Fresh frozen sections were thawed at room temperature and aired dried before fixation with 4% PFA/PBS for 30 min. Sections were washed followed by permeabilization with 0.3% Triton-X-100 in PBS (PBST) (15 min fixed frozen, 2 h optically cleared samples) before blocking (15-overnight minutes fixed frozen only) with 10% normal donkey or goat serum in PBST. Primary antibodies were diluted in PBST containing 2% normal serum and incubated with samples (overnight 4 °C for fixed frozen, 2–4 days room temperature with rocking for optically cleared samples) followed by PBST washes and incubation with secondary antibodies at room temperature (2 h fixed frozen, 1–2 days optical cleared) (Supplementary Table S3). Formalin fixed paraffin embedded sections were also used to test primary antibodies (human celiac ganglia, pancreas) using methods previously reported^71^. Omission of primary antibodies served as negative sample controls. Fixed frozen sections were mounted in Prolong Gold mounting media (ThermoFisher Scientific). PACT and iDISCO samples were mounted in refractive index matched solution (RIMS) (refractive index = 1.46) or Histo-2 (refractive index = 1.51) mounting media, respectively, before imaging^74^.

### Image acquisition, analysis, and quantification

Sample imaging was performed using a Zeiss 710 laser-scanning confocal microscope (Carl Zeiss). For Zeiss 710 image acquisition, a 10 × air objective (Zeiss Plan-Neofluar, 0.3 NA,), 20 × air objective (Zeiss Plan-Apochromat, 0.8 NA), 40 × air objective (Zeiss Plan-Apochromat, 0.95 NA) and a 63 × oil objective (Zeiss Plan-Apochromat, 1.4 NA) were used. Fixed frozen islets were imaged with z-stacks acquired. Optically cleared sections were tile-scanned with z-stacks acquired for a minimum of 100 µm depth with gain and offset optimized for the brightest central stack plane. Images were collected using 405, 488, 561, and 633 nm laser lines for excitation and emission spectra specific for the respective secondary antibodies (AF405/BV421, AF488, AF555, and AF647 fluorophores), with pinholes set to 1 airy unit for each channel, line averaging of 4, 8-bit, and 1024 × 1024 pixel image format.

The z-stacks were processed to obtain maximum intensity projections using Zen software (Carl Zeiss, https://www.zeiss.com/microscopy/us/products/microscope-software/zen.html). Islet NCAM and TH axon morphometry and density were analyzed using the neurite tracer program with ImageJ (NIH) and manual axons counts were performed by two investigators blinded to donor type. Optically cleared slices were analyzed using Neurolucida360 (MBF, https://www.mbfbioscience.com/neurolucida360) with contouring of islets and slices to define 3D volumes of exclusion and inclusion, respectively, and thresholding of TH-stained axons for morphometry (https://www.protocols.io/edit/human-pancreas-3d-image-analysis-using-neurolucida-bivwke7e). Intra-islet TH tracing was not performed due to lack of technical expertise for additional Neurolucida360 programming.

### Islet laser microdissection and RNA isolation

Serial fresh frozen sections (4 μm and 10 μm) were placed onto Superfrost Plus slides and polyethylene napthalate (PEN) membrane slides (Leica), respectively (Fig. 5a). The thin sections (4 μm) were stained by immunofluorescence for insulin (INS), glucagon (GCG), and CD3 to identify islets and T lymphocyte infiltration as previously reported^71^. Stained thin sections were imaged using the tile scan mode on a Zeiss710. Zeiss image files were converted to .tiff format and imported into Powerpoint software (Microsoft Office) to create reference maps for aiding islet identification in T1D donors as insulin negative (INS-) islets showed diminished autofluorescence. The thick 10 μm frozen sections were removed from − 80 °C storage and immediately dehydrated using 100% methanol, diethyl-pyrocarbonate-treated water, 75% ethanol, 95% ethanol, and two 100% ethanol steps (1 min each step except for 15 s rinse in water; all solutions kept cold on ice) followed by ≥ 5 min drying in a desiccator. Slides were loaded onto a Leica7000 microscope and islets were microdissected using a UV laser, 10 × objective/NA, and 50% power. Microdissected islets (~ 50/donor) dropped by gravity into RNAse-free caps of microvials (Fig. 5a). Islet RNA was immediately extracted using a PicoPure RNA kit with genomic DNA removal using recombinant DNase I (Worthington Biochemical Corporation). A 4 μl aliquot was made for RNA quality control (QC) assays and the remaining sample stored at − 80 °C until use. The QC assays for RNA concentration and quality (RIN) were determined using an Agilent 2100 Bioanalyzer (Picotape, Agilent Technologies) by the Genotyping and Gene Expression Analysis Core at the University of Florida Interdisciplinary Center for Biotechnology Research (ICBR).

### mRNA analysis by RNA-seq

Library construction and RNA-sequencing (RNA-seq) analysis were performed by the ICBR Core. Briefly, 6 ng RNA was used to extract mRNA with 15 μl of NEBNext Magnetic Oligo d(T) and fragmented in NEBNext First Strand Synthesis Buffer by heating at 94 °C for 8 min in a 96-well plate. First strand cDNA synthesis was performed using reverse transcriptase and random primers. Synthesis of double-stranded (ds) cDNA was performed using the second strand master mix provided in the kit. The resulting ds cDNA was end-repaired, dA-tailed, and ligated with NEBNext adaptors. The library was enriched by PCR amplification and purified by Agencourt AMPure beads (Beckman Coulter). Barcoded libraries were sized on the Bioanalyzer and quantitated by QUBIT. The library's functionality was validated by quantitative reverse transcription PCR (qRT-PCR), using the KAPA library quantification kit (Kapa Biosystems). The library was used to build clusters on the Illumina flow cell according to protocol that consists of 8 individual samples, pooled equimolarly for one lane of HiSeq 3000 sequencing. Paired-end, 100 cycle sequencing was performed on the Illumina HiSeq 3000 instrument using the clustering and sequencing reagents according to the manufacturer’s protocol. Under these run conditions, the cluster pass-filter was expected to be 70–75%, with a yield of 300–325 million pass-filter reads per lane. Greater than 95% of reads had ≥ Q30 score (Supplementary Fig. S2).

Islet transcriptome analysis was performed by the ICBR Bioinformatics Core. All RNA-seq reads were evaluated using fastqc^75^ and low quality reads were removed from downstream analysis using Trimmomatic^76^. High quality reads were aligned to human genome reference (hg38) using Bowtie2^77^ and quantitative gene expression was obtained using RSEM^78^. A multi-dimensional scaling (MDS) plot was used to detect outlier samples before differential gene expression analysis^79^. Empirical Bayes techniques were implemented using limma voom^80^ in R^81^ to identify differentially expressed genes between donor groups. Analyses were performed using Ingenuity Pathway Analysis software (Qiagen, https://digitalinsights.qiagen.com/products-overview/discovery-insights-portfolio/analysis-and-visualization/qiagen-ipa/) (see Supplementary Tables S4–5).

### Nanostring neuropathology panel

Islet RNA samples from 12 donors (n = 4/group) were analyzed using an RNA-based nCounter Elements assay by the NanoString Genomics facility (Human Neuropathology Panel, Nanostring Technologies, Seattle, WA, USA). Due to the small amount of RNA yielded by isolation from laser capture, the Nanostring Low Input kit was utilized. Briefly, 4 ng RNA was converted to cDNA, then amplified using primers specific to the 770 unique target sequences measured by the Human Neuropathology Panel, which was used to quantify the PCR product. A positive normalization factor was obtained using six internal reference standards in each of the 12 donor samples (1.01 ± 0.12). Eight negative controls were tested in each sample with a cut-off < 21 counts. Ten housekeeping genes were tested in each sample for further count normalization using a reference cut-off > 100 counts (Supplementary Fig. S5). Following QC procedures, 259 genes were removed for counts < 100. Data were analyzed by *t*-test using nCounter software (https://www.nanostring.com/products/ncounter-analysis-system/ncounter-systems-overview/) for significant differences between two pairs of donor groups, AAb versus ND and T1D versus ND according to manufacturer’s instructions (see Supplementary Tables S6–7).

### Statistics

Statistical analyses were performed with Excel (Microsoft Office) and GraphPad Prism 9.0 (GraphPad). Image analyses were performed by investigators blinded to donor group. Statistical methods included descriptive statistics and probability of normal distribution. Nonparametric tests were used for all datasets based on the D’Agostino & Pearson test for failure to pass normality test by at least 1 group (alpha = 0.05). Nested tables were created and a non-parametric 1-way ANOVA was applied to compare all groups after removal of outliers (ROUT method, Q = 0.5%). Adjusted p values are presented for Tukey’s multiple comparison tests. RNA-seq differential gene expression was determined by ANOVA and pairwise *t*-tests where p values were corrected for multiple testing using a Benjamini–Hochberg false discovery rate (FDR) set to an adjusted *P* < 0.05. For network and gene ontology analysis and Nanostring analyses, all statistical computations were performed by the corresponding packages. No statistical methods were used to predetermine sample size and our sample sizes are similar to those reported in previous publications. No method of randomization was used to determine how donors were allocated to groups due to matching for age, gender, and ethnicity as much as feasible.

Image analysis data are presented as scatterplots (mean, SD and individual points representing islets per donor. Nanostring gene expression data are presented as bar and whisker plots (median, range). Differences were considered significant at *P* < 0.05. Asterisks were used to indicate the significance level in figures: *0.01 P < 0.05.

### Ethical approval and consent to participate

The study protocol was reviewed and approved as nonhuman by the UF IRB, an independent ethics committee (IRB201902530). All animal procedures were performed in agreement with the published guidelines and regulations of the National Institutes of Health for the care and use of laboratory animals. The protocol was approved by the UF IACUC (IACUC 202009976). The authors confirm that all methods were carried out in accordance with relevant federal guidelines and regulations.

## Supplementary Information

Supplementary Information 1.

Supplementary Figures.

Supplementary Video 1.

Supplementary Video 2.

Supplementary Video 3.

## Data Availability

The datasets generated during and/or analyzed during the current study are available from the corresponding author on reasonable request. Accession numbers for the RNA-seq and Nanostring data reported in this paper in GEO: GSE (Pending). Original image files and data for NCAM1 and TH immunostaining in control donors are deposited in the NIH SPARC DAT-CORE Data Portal: https://doi.org/10.26275/ykxk-hasw.
