# Supplementary material for: Islet sympathetic innervation and islet neuropathology in patients with type 1 diabetes
Source: Sci Rep. 2021 Mar 22;11:6562. doi: 10.1038/s41598-021-85659-8 (PMC7985489; doi:10.1038/s41598-021-85659-8)
Supplement: Supplementary file 1 — Supplementary Information 1. [file 41598_2021_85659_MOESM1_ESM.docx]

Supplementary Materials for

**Islet Sympathetic Innervation and Islet Neuropathology in Patients with Type 1 Diabetes**

Martha Campbell-Thompson^*^, Elizabeth A. Butterworth, J. Lucas Boatwright, Malavika A. Nair, Lith H. Nasif, Kamal Nasif, Andy Y. Revell, Alberto Riva, Clayton E. Matthews, Ivan C. Gerling, Desmond A. Schatz, Mark A. Atkinson

*Corresponding author email: [mct@ufl.edu](mailto:mct@ufl.edu) (M.C-T)

**The PDF file includes:**

Tables S1-7

Legends for videos 1-3

**Other Supplementary Material for this manuscript includes the following:**

Videos 1-3

**Supplementary Table S1. Donor demographics.** Pancreas donor information for this study including sample use and Figures.

| Donor Type | CaseID | Age (years) | Diabetes Duration (years) | Gender | Race | BMI | C_peptide  (nl/ml) | HbA1c | Cause of Death | Islet Autoantibodies* | Assays | Fig |
| --- | --- | --- | --- | --- | --- | --- | --- | --- | --- | --- | --- | --- |
| No diabetes | 4001 | 7.00 |  | Male | Caucasian | 31.30 | NA | 5.50 | Head Trauma (MVA) | NA | OCT |  |
| No diabetes | 6007 | 9.00 |  | Male | African Am | 20.00 | NA |  | Anoxia | NA | OCT | 2a |
| No diabetes | 6293 | 9.00 |  | Female | Caucasian | 18.60 | 2.22 |  | Anoxia | Negative | RNAseq |  |
| No diabetes | 6318 | 10.00 |  | Female | Caucasian | 17.60 | 3.89 | 5.20 | Head Trauma | Negative | OCT |  |
| No diabetes | 6391 | 11.00 |  | Male | Caucasian | 19.00 | 4.23 | 5.30 | Anoxia | Negative | OCT |  |
| No diabetes | 6353 | 13.00 |  | Male | African Am | 28.30 | 1.76 | 5.40 | Anoxia | Negative | PACT |  |
| No diabetes | 6232 | 14.00 |  | Female | Caucasian | 20.80 | 1.12 |  | Head Trauma | Negative | Nanostring, RNAseq, PACT |  |
| No diabetes | 6374 | 14.00 |  | Female | Caucasian | 18.90 | 19.50 | 6.10 | Head Trauma | Negative | OCT |  |
| No diabetes | 6386 | 14.00 |  | Male | Caucasian | 23.90 | 13.42 | 5.60 | Head Trauma | Negative | OCT |  |
| No diabetes | 6099 | 14.20 |  | Male | Caucasian | 30.00 | 5.37 |  | Head Trauma | Negative | Nanostring |  |
| No diabetes | 6336 | 14.30 |  | Female | Caucasian | 28.90 | 7.87 | 5.20 | Head Trauma | Negative | OCT, RNAseq | 3a |
| No diabetes | 6372 | 14.40 |  | Male | African Am | 18.90 | 10.98 | 5.90 | Head Trauma | Negative | OCT |  |
| No diabetes | 6227 | 17.00 |  | Female | Caucasian | 26.40 | 2.75 |  | Cerebrovascular/Stroke | Negative | PACT |  |
| No diabetes | 6384 | 17.00 |  | Male | Caucasian | 18.20 | 0.70 | 4.80 | Head Trauma | Negative | OCT, PACT |  |
| No diabetes | 6098 | 17.80 |  | Male | Caucasian | 22.80 | 1.41 | 4.90 | Head Trauma | Negative | OCT |  |
| No diabetes | 6417 | 18.20 |  | Female | Caucasian | 24.50 | 3.94 | 5.10 | Head Trauma | Negative | iDISCO | 1d |
| No diabetes | 6389 | 18.60 |  | Male | Caucasian | 20.90 | 7.22 | 5.10 | Head Trauma | Negative | OCT |  |
| No diabetes | 6335 | 18.80 |  | Male | Multiracial | 23.60 | 8.85 | 5.30 | Head Trauma | Negative | PACT |  |
| No diabetes | 6279 | 19.00 |  | Male | Caucasian | 34.00 | 8.01 |  | Head Trauma | Negative | RNAseq, PACT | Video 3 |
| No diabetes | 6289 | 19.00 |  | Male | African Am | 38.30 | 8.05 |  | Head Trauma | Negative | OCT, PACT |  |
| No diabetes | 6234 | 20.00 |  | Female | Caucasian | 25.60 | 1.17 | 5.80 | Head Trauma (MVA) | Negative | OCT, RNAseq |  |
| No diabetes | 6238 | 20.00 |  | Male | African Am | 21.70 | 6.89 |  | Head Trauma | Negative | OCT, iDISCO, PACT | 1e |
| No diabetes | 6179 | 20.00 |  | Female | Caucasian | 20.70 | 2.74 |  | Head Trauma | Negative | OCT |  |
| No diabetes | 6160 | 22.10 |  | Male | Caucasian | 23.90 | 0.40 | 5.20 | Head Trauma | Negative | OCT |  |
| No diabetes | 6339 | 23.30 |  | Male | Caucasian | 25.00 | 10.56 | 5.30 | Head Trauma | Negative | RNAseq, PACT |  |
| No diabetes | 6131 | 24.20 |  | Male | Caucasian | 24.80 | 1.01 |  | Anoxia | Negative | Nanostring, RNAseq |  |
| No diabetes | 6401 | 25.07 |  | Female | Hispanic/Latino | 31.30 | 12.81 | 5.80 | Head Trauma (MVA) | Negative | Nanostring, RNAseq, iDISCO | 5a-e |
| No diabetes | 6126 | 25.20 |  | Male | Caucasian | 25.10 | 0.88 |  | Head Trauma | Negative | OCT |  |
| No diabetes | 6333 | 27.00 |  | Female | Caucasian | 24.90 | 9.37 | 4.70 | Anoxia | Negative | OCT |  |
| No diabetes | 4000 | 27.00 |  | Female | Caucasian | 20.00 |  | 5.10 | Cerebrovascular/Stroke | No serum available | iDISCO |  |
| No diabetes | 6229 | 31.00 |  | Female | Caucasian | 26.90 | 6.23 | 5.50 | Head Trauma (MVA) | Negative | OCT |  |
| No diabetes | 6251 | 33.00 |  | Female | Caucasian | 29.50 | 1.92 | 5.30 | Head Trauma | Negative | iDISCO |  |
| No diabetes | 6368 | 38.30 |  | Male | Caucasian | 20.70 | 3.05 | 5.20 | Head Trauma | Negative | OCT, PACT |  |
| No diabetes | 6290 | 58.00 |  | Male | Caucasian | 22.50 | 7.46 |  | Cerebrovascular/Stroke | Negative | OCT, PACT | 1E, Video 2 |
| AAb | 6424 | 17.65 |  | Male | Caucasian | 51.40 | 6.97 | 5.80 | Head Trauma | GADA+ mIAA+ | Nanostring, RNAseq |  |
| AAb | 6397 | 21.00 |  | Female | Caucasian | 29.60 | 1.49 | 6.00 | Head Trauma | GADA+ | OCT, iDISCO |  |
| AAb | 6314 | 21.00 |  | Male | Caucasian | 23.80 | 12.77 |  | Head Trauma (MVA) | GADA+ | OCT |  |
| AAb | 6429 | 22.10 |  | Male | African Am | 19.60 | 2.25 | 5.50 | Head Trauma (MVA) | GADA+ mIAA+ | Nanostring, RNAseq, iDISCO |  |
| AAb | 6267 | 23.00 |  | Female | Caucasian | 23.50 | 16.59 | 5.00 | Anoxia | GADA+ IA-2A+ | Nanostring, RNAseq, iDISCO |  |
| AAb | 6388 | 25.20 |  | Female | Hispanic/Latino | 26.00 | 1.38 | 5.70 | Anoxia | GADA+ mIAA+ | OCT | 1f |
| AAb | 6400 | 25.20 |  | Male | Hispanic/Latino | 22.20 | 4.17 | 5.50 | Head Trauma | GADA+ | OCT, iDISCO |  |
| AAb | 6301 | 26.00 |  | Male | African Am | 32.10 | 3.92 | 5.50 | Head Trauma | GADA+ | iDISCO |  |
| AAb | 6310 | 28.00 |  | Female | Hispanic/Latino | 22.40 | 10.54 |  | Anoxia | GADA+ | Nanostring |  |
| AAb | 6181 | 31.90 |  | Male | Caucasian | 21.90 | 0.06 |  | Head Trauma | GADA+ | OCT |  |
| T1D | 6209 | 5.00 | 0.25 | Female | Caucasian | 15.90 | 0.10 |  | DKA, cerebral edema | IA-2A+ ZnT8A+ mIAA+ | RNAseq |  |
| T1D | 6268 | 12.00 | 3 | Female | Caucasian | 26.56 | 0.18 | 9.80 | Anoxia | mIAA+ | Nanostring |  |
| T1D | 6052 | 12.00 | 1 | Male | African Am | 20.30 | 0.05 |  | DKA, cerebral edema | IA-2A+ mIAA+ | Nanostring |  |
| T1D | 6371 | 12.50 | 2 | Female | Caucasian | 16.60 | 0.11 | 9.50 | Cerebral edema | GADA+ IA-2A+ ZnT8A+ mIAA+ | RNAseq |  |
| T1D | 6228 | 13.00 | 0 | Male | Caucasian | 17.40 | 0.10 | 13.30 | Anoxia | GADA+ IA-2A+ ZnT8A+ | OCT, RNAseq, FFPE |  |
| T1D | 6113 | 13.10 | 1.58 | Female | Caucasian | 24.75 | 0.00 |  | Head Trauma | mIAA+ | Nanostring |  |
| T1D | 6342 | 14.00 | 2 | Female | Caucasian | 24.30 | 0.26 | 9.20 | Anoxia | IA-2A+ mIAA+ | RNAseq |  |
| T1D | 6089 | 14.30 | 8 | Male | Caucasian | 26.00 | 0.00 | 10.40 | Anoxia | mIAA+ | OCT |  |
| T1D | 6087 | 17.50 | 4 | Male | Caucasian | 21.90 | 0.00 |  | Head Trauma | ZnT8A+ mIAA+ | OCT |  |
| T1D | 6145 | 18.00 | 11 | Male | Caucasian | 23.10 | 0.06 |  | Head Trauma | GADA+ ZnT8A+ mIAA+ | OCT |  |
| T1D | 6306 | 19.00 | 5 | Male | Caucasian | 24.50 | 0.00 | 10.10 | Head Trauma | mIAA+ | RNAseq |  |
| T1D | 6296 | 20.00 | 12 | Male | Hispanic/Latino | 32.00 | 0.00 | 5.60 | Head Trauma | Negative | iDISCO |  |
| T1D | 6285 | 22.00 | 15 | Female | Hispanic/Latino | 21.80 | 0.00 |  | Head Trauma (MVA) | GADA+ IA-2A+ mIAA+ | iDISCO |  |
| T1D | 6330 | 22.00 | 18 | Male | Caucasian | 22.60 | 0.00 |  | Anoxia | IA-2A+ mIAA+ | OCT, iDISCO | 4b |
| T1D | 6414 | 23.10 | 0.43 | Male | African Am | 28.40 | 0.16 | 14.00 | Anoxia | GADA+ ZnT8A+ mIAA+ | RNAseq |  |
| T1D | 6362 | 24.90 | 0 | Male | Caucasian | 28.50 | 0.38 | 10.00 | Head Trauma | GADA+ | Nanostring, RNAseq |  |
| T1D | 6236 | 25.00 | 14 | Male | Caucasian | 20.10 | 0.00 | 11.60 | Anoxia | GADA+ mIAA+ | PACT, iDISCO |  |
| T1D | 6076 | 25.80 | 15 | Male | Caucasian | 18.80 | 0.00 | 8.20 | Anoxia | GADA+ mIAA+ | OCT |  |
| T1D | 6341 | 26.00 | 15 | Male | Caucasian | 21.80 | 0.00 | 13.60 | Cerebrovascular/Stroke | mIAA+ | PACT | 1c, 4a Video 1 |
| T1D | 6169 | 27.60 | 15 | Female | Hispanic/Latino | 25.00 | 0.00 | 9.50 | Anoxia | GADA+ mIAA+ | OCT |  |
| T1D | 6071 | 28.00 | 17 | Female | Caucasian | 19.5 | 0.00 | 7.3 | Pre-Existing Condition | IA-2A+ mIAA+ | FFPE | 1a |
| T1D | 6143 | 32.60 | 7 | Female | Caucasian | 26.10 | 0.00 |  | Anoxia | IA-2A+ mIAA+ | OCT |  |
| T2D | 6221 | 61.00 | 4 | Female | Caucasian | 33.70 | 3.05 |  | Cerebrovascular/Stroke | Negative | FFPE | 1b |

*No serum available (NA)

**Supplementary Table S2. Islet histopathology findings.** Fractional insulin area (FIA) and fractional glucagon area (FIA) determined in a former study are listed ^1^. Detection of islets with insulin+ β-cells (INS+) or without (INS-) determined by immunohistochemistry (IHC) as well as insulitis status are listed. Overall islet histopathological findings are listed. Additional histopathology information and access to IHC whole slide scans can be obtained by request for access to the nPOD Online Pathology database (email: nPOD@pathology.ufl.edu).

| Donor Type | CaseID | FIA* | FGA* | INS+ islets? | INS- islets? | Insulitis  detected | Islet Histopathology |
| --- | --- | --- | --- | --- | --- | --- | --- |
| No diabetes (ND) | 4001 |  |  | Yes | No | No | Ins+/Gluc+ normal islets. No significant findings. |
| No diabetes (ND) | 6007 | 1.00 |  | Yes | No | No | Ins+/Gluc+ normal islets. |
| No diabetes (ND) | 6293 | 2.36 | 1.54 | Yes | No | No | Ins+/Gluc+ normal numbers and morphology. Low Ki67 positivity in acinar cells and islets. |
| No diabetes (ND) | 6318 | 0.87 | 0.55 | Yes | No | No | Ins+/Gluc+ islets, normal sizes, morphologies, density. High exocrine Ki67 with only occasional islet with several Ki67+ cells. |
| No diabetes (ND) | 6391 |  |  | Yes | No | No | Ins+/Gluc+ islets, numerous, range of sizes and outlines. One 350um islet with reduced numbers of beta and alpha cells (PB 02). |
| No diabetes (ND) | 6353 |  |  | Yes | No | No | Ins+/Gluc+ islets, range of normal sizes with rare islet >500um. Moderate islet Ki67+. |
| No diabetes (ND) | 6386 |  |  | Yes | No | No | Ins+/Gluc islets, large range in morphologies and sizes with several very large islets (500-700um). Moderate islet nuclear pleomorphism. |
| No diabetes (ND) | 6232 | 1.79 | 1.03 | Yes | No | No | Ins+/Gluc+ islets, numerous. No significant findings. |
| No diabetes (ND) | 6374 |  |  | Yes | No | No | Ins+/Gluc+ islets, numerous, mostly small to medium sizes. Low islet and acinar cell Ki67+. |
| No diabetes (ND) | 6099 | 1.53 | 0.45 | Yes | No | No | Ins+/Gluc+ normal islets. |
| No diabetes (ND) | 6336 |  |  | Yes | No | No | Ins+/Gluc+ islets, normal size range and numbers. Sporadic increased Ki67+ cells/islets, primarily in tail region. |
| No diabetes (ND) | 6372 |  |  | Yes | No | No | Ins+/Gluc+ islets, numerous with range of normal sizes including several very large (>500um) islets. Moderate to high islet Ki67+ with focal increases in acinar Ki67+. |
| No diabetes (ND) | 6227 | 1.21 | 0.93 | Yes | No | No | Ins+/Gluc+ islets, no abnormalities observed. |
| No diabetes (ND) | 6384 |  |  | Yes | No | No | Ins+/Gluc+ islets, normal. Variable acinar and islet Ki67+ with low to moderate and moderate to very high, respectively. |
| No diabetes (ND) | 6098 | 0.54 | 0.36 | Yes | No | No | Ins+/Gluc+ normal islets, few with vascular stasis. |
| No diabetes (ND) | 6417 |  |  | Yes | No | No | Ins+/Gluc+ islets, wide range of densities, sizes and numbers including many single cells, clusters, small islets to several islets >500um. Variable numbers of Ki67+ cells within islets and low numbers in exocrine regions. |
| No diabetes (ND) | 6389 |  |  | Yes | No | No | Ins+/Gluc+ islets, within normal range of regional densities, sizes, and morphologies. |
| No diabetes (ND) | 6335 |  |  | Yes | No | No | Ins+/Gluc+ islets, numerous, range of normal sizes. Moderate to focally high islet and acinar cell Ki67+. |
| No diabetes (ND) | 6279 |  |  | Yes | No | No | Ins+/Gluc+ islet, numerous. Focal islet hyperplasia, PanTail 02. |
| No diabetes (ND) | 6289 |  |  | Yes | No | No | Ins+/Gluc+ islets, numerous. Ki67 slightly increased islets, increased acinar region. |
| No diabetes (ND) | 6238 | 1.83 | 0.62 | Yes | No | No | Ins+/Gluc+ islets, normal numbers. Low Ki67. |
| No diabetes (ND) | 6234 | 1.75 | 1.49 | Yes | No | No | Ins+/Gluc+ islets. No abnormalities observed. Low Ki67 all compartments. |
| No diabetes (ND) | 6179 |  | 0.28 | Yes | No | No | Ins+/Gluc+ islets, normal range of sizes, morphologies and density. Very high acinar Ki67. No other signification abnormalities observed. |
| No diabetes (ND) | 6160 | 0.51 | 0.59 | Yes | No | No | Ins+/Gluc+ islets present, all sizes. Low Ki67. |
| No diabetes (ND) | 6339 |  |  | Yes | No | No | Ins+/Gluc+ islets, normal morphologies and sizes including rare islets with reduced ratio beta to alpha cells. |
| No diabetes (ND) | 6131 |  |  | Yes | No | No | Ins+/Gluc+ islets. Occasional high islet Ki67. |
| No diabetes (ND) | 6401 |  |  | Yes | No | No | Ins+/Gluc+ islets, within normal range of numbers, sizes, and morphologies. Variable high islet and ductal epithelium Ki67+ cells. |
| No diabetes (ND) | 6126 | 0.83 | 0.43 | Yes | No | No | Ins+/Gluc+ islets, normal. Moderate Ki67 acinar cells and occasional islet. |
| No diabetes (ND) | 6333 |  |  | Yes | No | No | Ins+/Gluc+ islets, normal numbers and morphologies. |
| No diabetes (ND) | 4000 |  |  | Yes | No | No | Ins+/Gluc+ islets, normal range. |
| No diabetes (ND) | 6229 | 3.11 | 2.14 | Yes | No | No | Ins+/Gluc+ islets, no abnormalities observed. Occasional islet hyperemia. |
| No diabetes (ND) | 6251 | 1.84 | 1.11 | Yes | No | No | Ins+/Gluc+ islets, numerous, including single cells. Infrequent islet with 1-3 Ki67+ cells. No significant lesions. |
| No diabetes (ND) | 6368 |  |  | Yes | No | No | Ins+/Gluc+ islets, expected numbers and sizes with rare altered morphologies ("L", elongated). Variable very mild increase islet Ki67. Islet hyperemia (passive congestion) elevated body and tail regions. |
| No diabetes (ND) | 6290 |  |  | Yes | No | No | Ins+/Gluc+ islets, numerous. Vascular stasis observed within multiple islets (normal). |
| AAb | 6424 |  |  | Yes | No | No | Ins+/Gluc+ islets, normal range of sizes (including > 300um) and morphologies. Moderate increase islet and exocrine Ki67+ cell numbers. |
| AAb | 6314 |  |  | Yes | No | No | Ins+/Gluc+ islets, numerous, several large (200-400um). |
| AAb | 6397 |  |  | Yes | No | No | Ins+/Gluc+ islets, range of normal morphologies, sizes, and numbers. Very low acinar Ki67+. |
| AAb | 6429 |  |  | Yes | No | No | Ins+/Gluc+ islets, numerous with wide range sizes including several >500um and reduced ratios beta/alpha cells. Most islets have irregular contours. Rare large islet with 6 or more scattered CD3+ cells. No Ins- islets observed. No HLA hyperexpression observed. |
| AAb | 6267 | 1.98 | 1.39 | Yes | Yes | Yes | Ins+/Gluc+islets in normal numbers and density within regions except for focal islet hyperplasia in PanTail region (block 4). Insulitis found in all regions. Islets appear well demarcated with some having fibrosis. Pseudoatrophic islets. |
| AAb | 6388 |  |  | Yes | No | No | Ins+/Gluc+ islets, range of sizes and morphologies within normal limits. One possible INS- pseudoatrophic islet but additional levels same islet needed. |
| AAb | 6400 |  |  | Yes | No | No | Ins+/Gluc+ islets, range of sizes and morphologies including very large (500-700um). Variable islet Ki67+ levels, primarily low. |
| AAb | 6301 | 2.35 | 2.50 | Yes | No | No | Ins+/Gluc+ islets, prominent, numerous, some over 500um. |
| AAb | 6310 | 0.92 | 0.61 | Yes | Yes | Yes | Ins+/Gluc+ islets, numerous, some hyperplastic and >500um. Islet nuclear pleomorphism- mild. Insulitis- low grade, periphery and foci. Pseudoatrophic islets observed and other islets with low beta to alpha ratio. |
| AAb | 6181 | 0.55 | 0.26 | Yes | No | No | Ins+/Gluc+ islets, normal. |
| T1D | 6209 | 0.17 | 2.17 | Yes | Yes | Yes | Ins+ (reduced)/Gluc+ islets with insulitis. INS- islets present. Low Ki67 in all cell types. |
| T1D | 6052 | 0.02 | 1.32 | Yes | Yes | Yes | Ins+ (reduced numbers)/Gluc+ islets. INS- islets present. Insulitis, especially peri-islet. Heterogeneous islet distribution and size. Very high islet Ki67+. |
| T1D | 6268 | 0.25 | 2.39 | Yes | Yes | Yes | Ins+ (very rare)/Gluc+ islets, possibly reduced islet numbers but increased glucagon+ single cells. Insulitis present at insulin+ and insulin- islets. Ki67+ cells moderate numbers in acinar region, also in occasional islet. |
| T1D | 6371 |  |  | Yes | Yes | Yes | Ins+ (reduced numbers)/Gluc+ islets with widely varied sizes and overall reduced islet numbers. Insulitis: diffuse and/or aggregates of CD3+ cells at both insulin+ and insulin- islets. Moderate endocrine cell nuclear pleomorphism. Mild to focally moderate |
| T1D | 6228 | 0.34 | 1.45 | Yes | Yes | Yes | Ins+/Gluc+ Islets of various morphologies including normal appearing, fibrotic, degenerating, and with insulitis with infiltrate that includes eosinophils. Some pseudoatrophic (INS-) islets. |
| T1D | 6113 | 0.02 | 0.44 | Yes | Yes | Yes | Ins+ (reduced)/Gluc+ islets. Insulitis. Lobular islet heterogeneity. Islet atrophy with mild acinar atrophy (heterogeneous). Glucagon cells as single cells and small clusters. |
| T1D | 6342 |  |  | Yes | Yes | Yes | Ins+ (reduced numbers)/Gluc+, expected numbers, some with moderately increased sizes. Majority of islets are insulin- (pseudoatrophic) yet most blocks contain several insulin+ islets, with and without insulitis. Moderately increased Ki67 in both islets an |
| T1D | 6089 | 0.00 | 0.20 | No | Yes | No | Ins-/Gluc+ islets, small with irregular morphologies and decreased numbers (islet atrophy). |
| T1D | 6087 | 0.01 | 0.57 | No | Yes | No | Ins-/Gluc+ islets- reduced frequency. Mild, focal CD3+ islet infiltrates. |
| T1D | 6145 | 0.00 | 0.40 | No | Yes | No | Ins-/Gluc+ islets, atrophic. No infiltrates. |
| T1D | 6306 |  |  | Yes | Yes | Yes | Ins+/Gluc+ islets, reduced numbers but many are insulin+. Insulitis several islets, likely CD20+ as well as CD3+- note only some lymphocytes are CD3+. |
| T1D | 6296 | 0.12 | 1.25 | No | Yes | No | Ins-/Gluc+ islets (all pseudoatrophic), reduced numbers. |
| T1D | 6330 |  |  | No | Yes | No | Ins-/Gluc+ islets, reduced numbers. Flattened islet morphology typical of chronic T1D. Moderate exocrine atrophy. |
| T1D | 6285 |  |  | No | Yes | No | Ins-/Gluc+ islets, reduced numbers most regions. |
| T1D | 6414 |  |  | Yes | Yes | Yes | Ins+/Gluc+ islets (majority), small to large sized with primarily oval morphologies; single endocrine cells exocrine regions. Ins- islets and insulitis (both aggregate and diffuse types) present. |
| T1D | 6362 | 0.30 | 1.37 | Yes | Yes | Yes | Ins+/Gluc+ islets, moderate reduction numbers of Ins+ islets. Many islets with abnormal morphologies (large, fusing smaller islets, irregular outlines, fibrosis) in contrast to normal spherical/oblong islet morphologies. Insulitis in most Ins+ islets and some Ins- islets. |
| T1D | 6236 | 0.00 | 0.80 | No | Yes | No | Ins-/Gluc+ islets (reduced numbers). |
| T1D | 6076 | 0.00 | 0.72 | No | Yes | No | Ins- (reduced numbers)/Gluc+ islets. |
| T1D | 6341 |  |  | Yes | Yes | Yes | Ins-/Gluc+ (all pseudoatrophic), reduced numbers and sizes head and body regions. Insulitis found though with minimum numbers (6) of CD3+ cells; diffuse or in very small aggregates. |
| T1D | 6169 | 0.00 | 0.39 | No | Yes | No | Ins-/Gluc+ islets, marked atrophy. |
| T1D | 6071 |  |  | No | Yes | No | Ins-/Gluc+ islets. Few small islets that are present are Ins-/Gluc+/Som+. Amyloid. |
| T1D | 6143 | 0.00 | 0.94 | No | Yes | No | Ins-/Gluc + islets numerous. Extra-acinar islets. Foci of acinar degranulation often near islets. |
| T2D | 6221 |  |  | Yes | No | No | Ins+/Gluc+ islets, numerous, with amyloid. |

*Blank fields indicate data not available.

**Supplementary Table S3. Antibodies.** Primary and secondary antibodies used in this study.

| **Primary antibodies** | | |  | | |  | |  | | |  | | |  | |
| --- | --- | --- | --- | --- | --- | --- | --- | --- | --- | --- | --- | --- | --- | --- | --- |
| **Name** | **Antigen** | **Marker** | | | **Host** | | **Vendor** | | **Cat. #** | | | | **Dilution** | | **Link** |
| GCG | Glucagon | Alpha-cells (BV421 conjugated | | | Mouse | | BD Biosciences | | 565891 | | | | 200 | | <https://www.bdbiosciences.com/us/reagents/research/antibodies-buffers/cell-biology-reagents/cell-biology-antibodies/bv421-mouse-anti-glucagon-u16-850/p/565891> |
| GCG | Glucagon | Alpha-cells | | | Mouse | | Abcam | | ab10988 | | | | 500 | | <http://www.abcam.com/index.html?pageconfig=datasheet&intAbID=10988> |
| INS | Insulin | Beta-cells | | | Guinea Pig | | Dako | | A0564 | | | | 500 | | <https://www.agilent.com/en/product/immunohistochemistry/antibodies-controls/primary-antibodies/insulin-(autostainer-link-48)-76277> |
| NCAM | Neural cell adhesion molecular 1 | Neurons | | | Mouse | | Dako | | M730429-2 | | | | 200 | | https://www.agilent.com/store/en_US/Prod-M730429-2/M730429-2 |
| UCHL1 | Ubiquitin-protein hydrolase | Neurons | | | Rabbit | | Abcam | | ab108986 | | | | 200 | | <https://www.abcam.com/pgp95-antibody-epr4118-ab108986.html> |
| UCHL1 | Ubiquitin-protein hydrolase | Neurons | | | Chicken | | EnCor Bio | | CPCA-UCHL1 | | | | 200 | | <https://encorbio.com/product/cpca-uchl1/> |
| SCG3 | Secretogranin III | Neuroendocrine cells | | | Rabbit | | Sigma | | HPA006880 | | | | 500 | | https://www.sigmaaldrich.com/catalog/product/sigma/hpa006880?lang=en&region=US |
| SMA | Smooth muscle actin | Vascular myocytes | | | Mouse | | Sigma | | A5228 | | | | 200 | | <https://www.sigmaaldrich.com/catalog/product/sigma/a5228?lang=en&region=US> |
| SST | Somatostatin | Delta-cells | | | Goat | | Santa Cruz | | sc-7819 | | | | 500 | | <http://www.scbt.com/datasheet-7819-somatostatin-d-20-antibody.html> |
| SYN | Synapsin 1 | Synapse | | | Rabbit | | Thermofisher | | A-6442 | | | | 500 | | <https://www.thermofisher.com/antibody/product/A-6442.html?CID=AFLAP-A-6442> |
| SYP | Synaptophysin | Synapse | | | Mouse | | Sigma | | S5768 | | | | 200 | | <https://www.lsbio.com/antibodies/syp-antibody-synaptophysin-antibody-clone-oti3e2-if-immunofluorescence-ihc-wb-western-ls-c174787/182211> |
| TH | Tyrosine Hydroxylase | Sympathetic neurons | | | Rabbit | | Millipore | | AB152 | | | | 200 | | <http://www.emdmillipore.com/US/en/product/Anti-Tyrosine-Hydroxylase-Antibody,MM_NF-AB152> |
| TUBB3 | Beta-3 tubulin | Neurons | | | Mouse | | Abcam | | ab78078 | | | | 150 | | <https://www.abcam.com/beta-iii-tubulin-antibody-2g10-neuronal-marker-ab78078.html> |
|  |  |  | | |  | |  | |  | | | |  | |  |
|  |  |  | | |  | |  | |  | | | |  | |  |
| **Secondary antibodies** | | | |  | | |  | | |  | |  | | | |
|  | **Antigen** | **Fluorophore** | | | **Host** | | **Vendor** | | **Cat#** | | | | **Dilution** | | Link |
|  | Goat | AF633 | | | Donkey | | ThermoFisher Sci | | A-21082 | | | | 200-1000 | | <https://www.thermofisher.com/antibody/product/Donkey-anti-Goat-IgG-H-L-Cross-Adsorbed-Secondary-Antibody-Polyclonal/A-21082> |
|  | Mouse | AF488 | | | Donkey | | ThermoFisher Sci | | A-21202 | | | | 200-1000 | | <https://www.thermofisher.com/antibody/product/Donkey-anti-Mouse-IgG-H-L-Highly-Cross-Adsorbed-Secondary-Antibody-Polyclonal/A-21202> |
|  | Mouse | AF555 | | | Donkey | | ThermoFisher Sci | | A-31570 | | | | 200-1000 | | <http://www.thermofisher.com/antibody/product/A-31570.html?CID=AFLCA-A-31570> |
|  | Rabbit | AF488 | | | Donkey | | ThermoFisher Sci | | A-21206 | | | | 200-1000 | | <https://www.thermofisher.com/antibody/product/Donkey-anti-Rabbit-IgG-H-L-Highly-Cross-Adsorbed-Secondary-Antibody-Polyclonal/A-21206> |
|  | Rabbit | AF555 | | | Donkey | | ThermoFisher Sci | | A-31572 | | | | 200-1000 | | <http://www.thermofisher.com/antibody/product/A-31572.html?CID=AFLCA-A-31572> |
|  | Mouse | DyLight 405 | | | Goat | | ThermoFisher Sci | | 35500BID | | | | 200-1000 | | <https://www.thermofisher.com/antibody/product/Goat-anti-Mouse-IgG-H-L-Cross-Adsorbed-Secondary-Antibody-Polyclonal/35500BID> |
|  | Rabbit | AF633 | | | Goat | | ThermoFisher Sci | | A-21082 | | | | 200-1000 | | <https://www.thermofisher.com/antibody/product/Donkey-anti-Goat-IgG-H-L-Cross-Adsorbed-Secondary-Antibody-Polyclonal/A-21082> |
|  | Guinea pig | AF488 | | | Goat | | ThermoFisher Sci | | A-11073 | | | | 200-1000 | | <https://www.thermofisher.com/antibody/product/Goat-anti-Guinea-Pig-IgG-H-L-Highly-Cross-Adsorbed-Secondary-Antibody-Polyclonal/A-11073> |
|  | Chicken | DyLight 488 | | | Goat | | ThermoFisher Sci | | SA5-10070 | | | | 200-500 | | <https://www.thermofisher.com/antibody/product/Goat-anti-Chicken-IgY-H-L-Cross-Adsorbed-Secondary-Antibody-Polyclonal/SA5-10070?pluginName=> |

**Supplementary Table S4. RNA-seq Canonical Pathways.** Top canonical pathways are shown between T1D vs ND (green), AAb-T1D (yellow), and AAb-ND (blue) comparisons.

| Analysis | Ingenuity Canonical Pathways | -log(p-value) | Ratio | Molecules |
| --- | --- | --- | --- | --- |
| T1D-ND | Noradrenaline and Adrenaline Degradation | 0.00 | 2.86E-02 | DHRS |
| T1D-ND | Dopamine Receptor Signaling | 0.00 | 2.63E-02 | TH,PPP2CA |
| T1D-ND | α-Adrenergic Signaling | 0.00 | 2.35E-02 | GNAS,PRKCB |
| T1D-ND | Serotonin Degradation | 0.00 | 1.54E-02 | DHRS2 |
| T1D-ND | GABA Receptor Signaling | 0.30 | 4.26E-02 | KCNQ2,CACNA2D4,GNAS,GABRE |
| T1D-ND | Synaptic Long Term Potentiation | 0.31 | 4.20E-02 | PPP1R1A,PLCB4,PRKCB,PDIA3,GRIA4 |
| T1D-ND | Cardiac β-adrenergic Signaling | 0.34 | 4.29E-02 | GNAS,PDE7A,PDE6A,PPP1R1A,PKIB,PPP2CA |
| T1D-ND | Dopamine-DARPP32 Feedback in cAMP Signaling | 0.37 | 4.37E-02 | CREM,GNAS,GUCY1B1,PLCB4,PRKCB,PDIA3,PPP2CA |
| T1D-ND | Glutamate Receptor Signaling | 0.43 | 5.36E-02 | GRIK1,SLC17A6,GRIA4 |
| T1D-ND | Synaptic Long Term Depression | 0.69 | 5.36E-02 | CACNA2D4,GNAS,GUCY1B1,PLCB4,PRKCB,PDIA3,PNPLA8,GRIA4,PPP2CA |
| T1D-ND | Catecholamine Biosynthesis | 0.84 | 2.50E-01 | TH |
| T1D-ND | Type II Diabetes Mellitus Signaling | 0.86 | 5.92E-02 | FGFR3,FGFR2,CACNA2D4,SLC2A2,PRKCB,PIK3R5,MAFA,SOCS2,PDX1 |
| T1D-ND | Neuroinflammation Signaling Pathway | 0.90 | 5.32E-02 | FGFR3,FGFR2,HLA-DOA,CCL5,TGFBR3,PIK3R5,HLA-DOB,BIRC3,HLA-DRA,NCF2,HLA-DRB1,HLA-DMA,JMJD7-PLA2G4B,HLA-B,GABRE,CD200 |
| T1D-ND | nNOS Signaling in Neurons | 0.99 | 8.70E-02 | RASD1,PRKCB,PFKM,DLG2 |
| T1D-ND | Neuroprotective Role of THOP1 in Alzheimer's Disease | 1.16 | 7.14E-02 | PRSS8,HLA-E,GZMA,TMPRSS2,KLK11,HLA-B,HLA-F,PRSS23 |
| T1D-ND | Neuropathic Pain Signaling In Dorsal Horn Neurons | 1.48 | 7.89E-02 | FGFR3,FGFR2,KCNQ2,SRC,PLCB4,PRKCB,PIK3R5,PDIA3,GRIA4 |
| T1D-ND | Parkinson's Signaling | 1.65 | 1.88E-01 | PARK7,UCHL1,CYCS |
| T1D-ND | Axonal Guidance Signaling | 1.81 | 6.05E-02 | FGFR2,SEMA4G,C9orf3,BMP5,PIK3R5,TUBA1A,ADAMTS5,ERBB2,PRKCB,PSMD14,UNC5D,STK36,BMP7,FGFR3,PLCB4,ROBO1,UNC5A,RTN4,PDGFC,GNAS,SHANK2,MKNK1,TUBA1B,PDIA3,EIF4E,PFN2,EPHA8 |
| T1D-ND | Maturity Onset Diabetes of Young (MODY) Signaling | 3.08 | 2.50E-01 | SLC2A2,HNF4A,GAPDH,HNF1B,PDX1 |
|  |  |  |  |  |
| AAb-T1D | CXCR4 Signaling | 1.61 | 6.10E-03 | ELMO1 |
|  |  |  |  |  |
| AAb-ND | Glucocorticoid Receptor Signaling | 1.01 | 2.98E-03 | KRT17 |
| AAb-ND | B Cell Receptor Signaling | 1.25 | 5.32E-03 | IGHA1 |
| AAb-ND | Communication between Innate and Adaptive Immune Cells | 1.58 | 1.15E-02 | IGHA1 |
| AAb-ND | Primary Immunodeficiency Signaling | 1.87 | 2.27E-02 | IGHA1 |
| AAb-ND | MIF Regulation of Innate Immunity | 1.88 | 2.33E-02 | CD74 |
| AAb-ND | Hematopoiesis from Pluripotent Stem Cells | 1.89 | 2.38E-02 | IGHA1 |
| AAb-ND | Antigen Presentation Pathway | 1.94 | 2.63E-02 | CD74 |
| AAb-ND | MIF-mediated Glucocorticoid Regulation | 1.97 | 2.86E-02 | CD74 |

**Supplementary Table S5. RNA-seq Differential Gene Expression between groups.** Top differentially expressed genes for T1D vs ND (N=100, green), AAb-ND (blue) and AAb-T1D (yellow) are shown determined by pair-wise t-tests.

| T1D-ND | **Expr Log Ratio** | **Expr p-value** |  | AAb-ND | **Expr Log Ratio** | **Expr p-value** |  | AAb-T1D | **Expr Log Ratio** | **Expr p-value** |
| --- | --- | --- | --- | --- | --- | --- | --- | --- | --- | --- |
| MT1G | 2.88 | 1.40E-04 |  | CD74 | 2.14 | 3.88E-02 |  | ELMO1 | 2.86 | 1.83E-02 |
| ELMO1 | -2.86 | 2.95E-04 |  | IGHA1 | 8.68 | 3.28E-02 |  | GLRA1 | 5.62 | 3.92E-02 |
| CD74 | 2.23 | 5.00E-04 |  | KRT17 | 6.60 | 4.35E-02 |  | MT1G | -2.68 | 1.83E-02 |
| ENTPD3 | -3.18 | 1.17E-03 |  | LAG3 | 5.05 | 3.88E-02 |  |  |  |  |
| HADH | -2.51 | 1.17E-03 |  | STYK1 | 5.36 | 3.88E-02 |  |  |  |  |
| HLA-F | 1.73 | 1.17E-03 |  | VSTM5 | 5.04 | 4.35E-02 |  |  |  |  |
| PTPN22 | 5.18 | 1.17E-03 |  |  |  |  |  |  |  |  |
| PKIB | -2.55 | 2.44E-03 |  |  |  |  |  |  |  |  |
| ARG2 | -3.00 | 2.49E-03 |  |  |  |  |  |  |  |  |
| PFKFB2 | -2.84 | 3.01E-03 |  |  |  |  |  |  |  |  |
| HHATL | -7.46 | 3.26E-03 |  |  |  |  |  |  |  |  |
| MYCN | -5.30 | 3.26E-03 |  |  |  |  |  |  |  |  |
| IAPP | -9.57 | 4.14E-03 |  |  |  |  |  |  |  |  |
| ADCYAP1 | -4.68 | 5.46E-03 |  |  |  |  |  |  |  |  |
| ERMN | -7.09 | 5.80E-03 |  |  |  |  |  |  |  |  |
| SFRP5 | 2.23 | 5.80E-03 |  |  |  |  |  |  |  |  |
| APOC1 | 3.03 | 5.87E-03 |  |  |  |  |  |  |  |  |
| C1GALT1C1 | -1.18 | 5.87E-03 |  |  |  |  |  |  |  |  |
| HTR7P1 | 3.85 | 5.87E-03 |  |  |  |  |  |  |  |  |
| PTPRQ | 5.51 | 5.87E-03 |  |  |  |  |  |  |  |  |
| SEC11C | -1.72 | 5.87E-03 |  |  |  |  |  |  |  |  |
| SLC2A2 | -3.85 | 5.87E-03 |  |  |  |  |  |  |  |  |
| CD7 | 3.90 | 6.83E-03 |  |  |  |  |  |  |  |  |
| DLK1 | -6.82 | 6.83E-03 |  |  |  |  |  |  |  |  |
| HAPLN4 | -5.87 | 6.83E-03 |  |  |  |  |  |  |  |  |
| HLA-DRA | 1.72 | 6.83E-03 |  |  |  |  |  |  |  |  |
| KLRC4-KLRK1/KLRK1 | 5.24 | 6.83E-03 |  |  |  |  |  |  |  |  |
| KRT7 | 2.67 | 6.83E-03 |  |  |  |  |  |  |  |  |
| NME7 | -1.44 | 6.83E-03 |  |  |  |  |  |  |  |  |
| PPP1R1A | -3.51 | 6.83E-03 |  |  |  |  |  |  |  |  |
| PRDX3 | -1.28 | 6.83E-03 |  |  |  |  |  |  |  |  |
| RNF6 | -1.48 | 6.83E-03 |  |  |  |  |  |  |  |  |
| SELENOF | -1.19 | 6.83E-03 |  |  |  |  |  |  |  |  |
| WSCD2 | -5.12 | 6.83E-03 |  |  |  |  |  |  |  |  |
| TVP23B | -1.32 | 6.97E-03 |  |  |  |  |  |  |  |  |
| OSTC | -1.33 | 7.09E-03 |  |  |  |  |  |  |  |  |
| FAM105A | -1.80 | 7.17E-03 |  |  |  |  |  |  |  |  |
| TRPM3 | -2.02 | 7.17E-03 |  |  |  |  |  |  |  |  |
| KLHDC8A | -5.02 | 7.89E-03 |  |  |  |  |  |  |  |  |
| KLHL1 | -6.22 | 7.89E-03 |  |  |  |  |  |  |  |  |
| SRP9 | -0.95 | 7.89E-03 |  |  |  |  |  |  |  |  |
| NPTX2 | -2.55 | 7.97E-03 |  |  |  |  |  |  |  |  |
| GLRA1 | -4.81 | 8.64E-03 |  |  |  |  |  |  |  |  |
| APOD | 2.20 | 8.81E-03 |  |  |  |  |  |  |  |  |
| TSPAN13 | -1.46 | 8.81E-03 |  |  |  |  |  |  |  |  |
| CYP2U1 | -1.78 | 8.83E-03 |  |  |  |  |  |  |  |  |
| NUCB2 | -1.06 | 8.83E-03 |  |  |  |  |  |  |  |  |
| SRD5A1 | -2.05 | 8.83E-03 |  |  |  |  |  |  |  |  |
| WLS | -1.21 | 8.83E-03 |  |  |  |  |  |  |  |  |
| FGD2 | 4.07 | 9.04E-03 |  |  |  |  |  |  |  |  |
| ERLEC1 | -1.12 | 9.04E-03 |  |  |  |  |  |  |  |  |
| LRRC42 | -1.68 | 9.04E-03 |  |  |  |  |  |  |  |  |
| SAMD11 | -3.31 | 9.04E-03 |  |  |  |  |  |  |  |  |
| SCNN1A | 1.87 | 9.04E-03 |  |  |  |  |  |  |  |  |
| AIG1 | -1.03 | 9.28E-03 |  |  |  |  |  |  |  |  |
| HNF1B | 1.45 | 9.28E-03 |  |  |  |  |  |  |  |  |
| SCD5 | -1.48 | 9.28E-03 |  |  |  |  |  |  |  |  |
| ARL1 | -1.09 | 9.36E-03 |  |  |  |  |  |  |  |  |
| SERPING1 | 1.65 | 9.36E-03 |  |  |  |  |  |  |  |  |
| DACH2 | -4.22 | 9.56E-03 |  |  |  |  |  |  |  |  |
| RDH11 | -1.02 | 9.67E-03 |  |  |  |  |  |  |  |  |
| STK17B | 2.24 | 1.04E-02 |  |  |  |  |  |  |  |  |
| CACNA2D4 | 3.82 | 1.05E-02 |  |  |  |  |  |  |  |  |
| CHN1 | -1.82 | 1.05E-02 |  |  |  |  |  |  |  |  |
| CLEC2D | 1.55 | 1.05E-02 |  |  |  |  |  |  |  |  |
| LRRTM3 | -3.45 | 1.05E-02 |  |  |  |  |  |  |  |  |
| RAB1A | -0.88 | 1.05E-02 |  |  |  |  |  |  |  |  |
| CABP7 | -2.47 | 1.11E-02 |  |  |  |  |  |  |  |  |
| ARF4 | -1.22 | 1.11E-02 |  |  |  |  |  |  |  |  |
| GHITM | -1.09 | 1.11E-02 |  |  |  |  |  |  |  |  |
| HLA-DOB | 5.59 | 1.11E-02 |  |  |  |  |  |  |  |  |
| KCNG3 | -4.42 | 1.11E-02 |  |  |  |  |  |  |  |  |
| ACTR3B | -1.78 | 1.13E-02 |  |  |  |  |  |  |  |  |
| PCSK1 | -2.81 | 1.13E-02 |  |  |  |  |  |  |  |  |
| PRPS1 | -1.52 | 1.13E-02 |  |  |  |  |  |  |  |  |
| RRAGA | -0.82 | 1.13E-02 |  |  |  |  |  |  |  |  |
| SYT13 | -2.17 | 1.13E-02 |  |  |  |  |  |  |  |  |
| BET1 | -0.96 | 1.13E-02 |  |  |  |  |  |  |  |  |
| DEUP1 | -3.33 | 1.13E-02 |  |  |  |  |  |  |  |  |
| MDH2 | -1.41 | 1.13E-02 |  |  |  |  |  |  |  |  |
| MMP1 | 5.03 | 1.13E-02 |  |  |  |  |  |  |  |  |
| MYRF | 1.22 | 1.13E-02 |  |  |  |  |  |  |  |  |
| PARM1 | -1.31 | 1.13E-02 |  |  |  |  |  |  |  |  |
| PTPRC | 2.21 | 1.13E-02 |  |  |  |  |  |  |  |  |
| SOCS2 | -1.40 | 1.13E-02 |  |  |  |  |  |  |  |  |
| TUBA1A | -1.90 | 1.13E-02 |  |  |  |  |  |  |  |  |
| OR13A1 | -4.71 | 1.13E-02 |  |  |  |  |  |  |  |  |
| SP5 | -3.27 | 1.13E-02 |  |  |  |  |  |  |  |  |
| LMNTD1 | -4.64 | 1.16E-02 |  |  |  |  |  |  |  |  |
| AMPH | -2.44 | 1.16E-02 |  |  |  |  |  |  |  |  |
| CASR | -1.59 | 1.16E-02 |  |  |  |  |  |  |  |  |
| H2AFZ | -0.86 | 1.16E-02 |  |  |  |  |  |  |  |  |
| HLA-B | 1.44 | 1.16E-02 |  |  |  |  |  |  |  |  |
| HMCN1 | 5.13 | 1.16E-02 |  |  |  |  |  |  |  |  |
| KRT17 | 5.49 | 1.16E-02 |  |  |  |  |  |  |  |  |
| OCIAD1 | -1.07 | 1.16E-02 |  |  |  |  |  |  |  |  |
| PATL2 | 4.68 | 1.16E-02 |  |  |  |  |  |  |  |  |
| RAB2A | -0.88 | 1.16E-02 |  |  |  |  |  |  |  |  |
| SAR1B | -1.11 | 1.16E-02 |  |  |  |  |  |  |  |  |

**Supplementary Table S6. Nanostring differential gene expression T1D vs. ND.** Top 100 differentially expressed genes between T1D vs. ND by Nanostring.

| Gene | Log2 fold change | P-value | Unadj.  p.value | Gene.sets |
| --- | --- | --- | --- | --- |
| HLA-DRA | 3.14 | 8.25E-05 | 8.25E-05 | Disease Association, Tissue Integrity |
| SP100 | 1.14 | 3.37E-04 | 3.37E-04 | Angiogenesis |
| TRIM37 | -0.959 | 3.41E-04 | 3.41E-04 | Chromatin Modification, Unfolded Protein Response |
| TLR2 | 1.81 | 5.19E-04 | 5.19E-04 | Activated Microglia, Disease Association |
| ATP6V1D | -0.728 | 1.32E-03 | 1.32E-03 | Disease Association, Transmitter Synthesis and Storage |
| PPP2CA | -0.489 | 1.44E-03 | 1.44E-03 | Carbohydrate Metabolism, Disease Association, Transmitter Release, Transmitter Response and Reuptake |
| IL10RA | 1.28 | 1.50E-03 | 1.50E-03 | Cytokines, Disease Association |
| AMPH | -1.62 | 1.51E-03 | 1.51E-03 | Neural Connectivity, Vesicle Trafficking |
| PSMB9 | 2.43 | 1.57E-03 | 1.57E-03 | Activated Microglia, Disease Association, Unfolded Protein Response |
| GFPT1 | -1.52 | 1.65E-03 | 1.65E-03 | Unfolded Protein Response |
| RTN4 | -1.3 | 1.72E-03 | 1.72E-03 | Disease Association, Growth Factor Signaling |
| GRIA4 | -2.25 | 2.09E-03 | 2.09E-03 | Axon and Dendrite Structure, Neural Connectivity, Transmitter Release, Transmitter Response and Reuptake, Vesicle Trafficking |
| TAF4 | 0.773 | 2.25E-03 | 2.25E-03 | Disease Association, Transcription and Splicing |
| LPAR1 | 1.58 | 2.49E-03 | 2.49E-03 | Axon and Dendrite Structure, Myelination, Neural Connectivity, Transmitter Response and Reuptake |
| CTNS | 1.04 | 2.78E-03 | 2.78E-03 | Autophagy |
| CASP1 | 1.86 | 2.83E-03 | 2.83E-03 | Apoptosis, Disease Association |
| SLC2A1 | -1.38 | 2.86E-03 | 2.86E-03 | Activated Microglia, Disease Association, Tissue Integrity |
| PGAM1 | -0.79 | 3.28E-03 | 3.28E-03 | Carbohydrate Metabolism |
| DGKB | -1.98 | 3.42E-03 | 3.42E-03 | Lipid Metabolism |
| RAPGEF2 | 0.475 | 3.43E-03 | 3.43E-03 | Growth Factor Signaling, Neural Connectivity, Vesicle Trafficking |
| CAMK2G | 0.693 | 3.46E-03 | 3.46E-03 | Disease Association, Growth Factor Signaling, Transmitter Release, Transmitter Response and Reuptake, Trophic Factors |
| GUCY1B3 | -1.02 | 3.65E-03 | 3.65E-03 | Oxidative Stress, Transmitter Response and Reuptake |
| POLR2B | -0.465 | 3.67E-03 | 3.67E-03 | Disease Association, Transcription and Splicing |
| ADCYAP1 | -2.47 | 4.47E-03 | 4.47E-03 | Axon and Dendrite Structure, Disease Association, Neural Connectivity, Transmitter Synthesis and Storage, Vesicle Trafficking |
| GNAI3 | -0.616 | 4.53E-03 | 4.53E-03 | Disease Association, Transmitter Release, Transmitter Response and Reuptake |
| RAN | -0.583 | 5.30E-03 | 5.30E-03 | Disease Association, Transcription and Splicing |
| CYBB | 1.8 | 5.55E-03 | 5.55E-03 | Activated Microglia, Disease Association, Oxidative Stress |
| FAS | 1.27 | 5.70E-03 | 5.70E-03 | Apoptosis, Axon and Dendrite Structure, Cytokines, Disease Association, Growth Factor Signaling, Oxidative Stress |
| ERBB3 | 1.47 | 6.08E-03 | 6.08E-03 | Growth Factor Signaling, Transmitter Release |
| STAT3 | -0.587 | 6.53E-03 | 6.53E-03 | Disease Association, Growth Factor Signaling |
| MYRF | 0.845 | 6.66E-03 | 6.66E-03 | Myelination |
| CD40 | 0.947 | 7.41E-03 | 7.41E-03 | Cytokines, Tissue Integrity |
| STAT1 | 1.53 | 7.50E-03 | 7.50E-03 | Activated Microglia, Angiogenesis, Axon and Dendrite Structure, Growth Factor Signaling |
| PSMB8 | 1.05 | 7.81E-03 | 7.81E-03 | Activated Microglia, Disease Association, Unfolded Protein Response |
| PDE1B | -1.78 | 8.28E-03 | 8.28E-03 | Disease Association, Transmitter Release, Transmitter Synthesis and Storage |
| PGK1 | -0.474 | 8.34E-03 | 8.34E-03 | Activated Microglia, Carbohydrate Metabolism |
| RAB2A | -0.649 | 9.27E-03 | 9.27E-03 | Carbohydrate Metabolism |
| UCHL1 | -1.52 | 9.46E-03 | 9.46E-03 | Axon and Dendrite Structure, Disease Association |
| MAPKAPK2 | -0.718 | 9.81E-03 | 9.81E-03 | Growth Factor Signaling, Trophic Factors |
| CD68 | 1.73 | 9.94E-03 | 9.94E-03 | Activated Microglia, Autophagy, Disease Association |
| TAF9 | -0.851 | 1.07E-02 | 1.07E-02 | Chromatin Modification, Transcription and Splicing |
| CNTN1 | -0.826 | 1.29E-02 | 1.29E-02 | Axon and Dendrite Structure, Disease Association, Tissue Integrity |
| GLRB | -0.565 | 1.33E-02 | 1.33E-02 | Axon and Dendrite Structure, Neural Connectivity, Transmitter Release, Transmitter Response and Reuptake, Vesicle Trafficking |
| ITGAM | 2.53 | 1.45E-02 | 1.45E-02 | Disease Association, Tissue Integrity |
| CPT1B | 1.08 | 1.66E-02 | 1.66E-02 | Carbohydrate Metabolism |
| DLD | -0.451 | 1.72E-02 | 1.72E-02 | Carbohydrate Metabolism |
| NOTCH1 | 1.21 | 1.89E-02 | 1.89E-02 | Angiogenesis, Disease Association, Growth Factor Signaling |
| CX3CL1 | 1.41 | 1.94E-02 | 1.94E-02 | Angiogenesis, Cytokines, Neural Connectivity, Vesicle Trafficking |
| GTF2IRD1 | -1.01 | 1.94E-02 | 1.94E-02 | Transcription and Splicing, Transmitter Response and Reuptake |
| SPTBN2 | -0.43 | 1.95E-02 | 1.95E-02 | Disease Association, Neural Connectivity, Neuronal Cytoskeleton |
| SLU7 | -0.643 | 2.11E-02 | 2.11E-02 | Transcription and Splicing |
| EMP2 | -1.2 | 2.20E-02 | 2.20E-02 | Angiogenesis |
| INPP5F | -1.17 | 2.46E-02 | 2.46E-02 | Growth Factor Signaling |
| BCHE | -1.12 | 2.49E-02 | 2.49E-02 | Carbohydrate Metabolism, Disease Association, Vesicle Trafficking |
| CREBBP | 0.384 | 2.61E-02 | 2.61E-02 | Chromatin Modification, Disease Association, Growth Factor Signaling, Transmitter Response and Reuptake |
| PRKACA | -0.661 | 2.69E-02 | 2.69E-02 | Disease Association, Growth Factor Signaling, Neural Connectivity, Transmitter Release, Transmitter Response and Reuptake, Vesicle Trafficking |
| AIF1 | 1.87 | 2.71E-02 | 2.71E-02 | Disease Association, Oxidative Stress |
| TENM2 | 1.19 | 2.71E-02 | 2.71E-02 | Axon and Dendrite Structure, Neural Connectivity |
| PARK7 | -0.374 | 2.87E-02 | 2.87E-02 | Axon and Dendrite Structure, Disease Association, Neural Connectivity, Oxidative Stress, Transmitter Release, Transmitter Synthesis and Storage, Vesicle Trafficking |
| PLA2G6 | 0.782 | 2.96E-02 | 2.96E-02 | Lipid Metabolism, Vesicle Trafficking |
| BECN1 | -0.326 | 2.99E-02 | 2.99E-02 | Axon and Dendrite Structure |
| EGF | 1.82 | 3.21E-02 | 3.21E-02 | Angiogenesis, Cytokines, Growth Factor Signaling |
| KIAA1161 | 0.972 | 3.24E-02 | 3.24E-02 |  |
| SHANK2 | 1.17 | 3.25E-02 | 3.25E-02 | Axon and Dendrite Structure, Neural Connectivity, Transmitter Release, Vesicle Trafficking |
| CCNH | -0.362 | 3.27E-02 | 3.27E-02 | Transcription and Splicing |
| ATP6V1A | -0.505 | 3.34E-02 | 3.34E-02 | Activated Microglia, Transmitter Synthesis and Storage |
| DNAH1 | 1.06 | 3.36E-02 | 3.36E-02 | Disease Association, Neuronal Cytoskeleton |
| HDAC6 | -0.457 | 3.38E-02 | 3.38E-02 | Axon and Dendrite Structure, Chromatin Modification, Oxidative Stress |
| MAPK8 | -0.372 | 3.38E-02 | 3.38E-02 | Apoptosis, Chromatin Modification, Disease Association, Growth Factor Signaling, Transmitter Release, Transmitter Response and Reuptake, Trophic Factors, Unfolded Protein Response |
| SLC4A10 | -0.94 | 3.43E-02 | 3.43E-02 | Axon and Dendrite Structure |
| EFNA5 | -0.835 | 3.48E-02 | 3.48E-02 | Growth Factor Signaling, Tissue Integrity |
| SIRT7 | 0.871 | 3.55E-02 | 3.55E-02 | Chromatin Modification |
| SYNJ1 | -0.487 | 3.55E-02 | 3.55E-02 | Axon and Dendrite Structure, Disease Association, Growth Factor Signaling, Neural Connectivity, Vesicle Trafficking |
| INSR | 0.713 | 3.82E-02 | 3.82E-02 | Carbohydrate Metabolism, Neural Connectivity, Transmitter Response and Reuptake |
| ADORA2A | 1.07 | 3.84E-02 | 3.84E-02 | Disease Association, Transmitter Release, Transmitter Response and Reuptake |
| CSF1 | 1.08 | 3.89E-02 | 3.89E-02 | Activated Microglia, Cytokines |
| CASP7 | 0.939 | 3.92E-02 | 3.92E-02 | Apoptosis, Disease Association |
| ERLEC1 | -0.639 | 3.97E-02 | 3.97E-02 | Unfolded Protein Response |
| RASGRP1 | -0.828 | 4.26E-02 | 4.26E-02 | Growth Factor Signaling |
| ITGA7 | 0.837 | 4.28E-02 | 4.28E-02 | Tissue Integrity |
| ADCY5 | 1.33 | 4.30E-02 | 4.30E-02 | Disease Association, Transmitter Release, Transmitter Response and Reuptake |
| BCL2 | 1.81 | 4.31E-02 | 4.31E-02 | Activated Microglia, Apoptosis, Disease Association, Growth Factor Signaling, Oxidative Stress, Transmitter Release, Trophic Factors, Unfolded Protein Response |
| ADAM10 | 0.626 | 4.32E-02 | 4.32E-02 | Disease Association, Neural Connectivity |
| SQSTM1 | 0.281 | 4.37E-02 | 4.37E-02 | Autophagy, Disease Association |
| TBPL1 | -0.581 | 4.52E-02 | 4.52E-02 | Disease Association, Transcription and Splicing |
| ITPR1 | 0.806 | 4.58E-02 | 4.58E-02 | Apoptosis, Disease Association, Neural Connectivity, Transmitter Release, Transmitter Response and Reuptake |
| ATCAY | 0.765 | 4.71E-02 | 4.71E-02 | Axon and Dendrite Structure, Neural Connectivity |
| ATP6V0E1 | -0.477 | 4.82E-02 | 4.82E-02 | Activated Microglia, Transmitter Synthesis and Storage |
| CUL3 | -0.267 | 4.82E-02 | 4.82E-02 | Growth Factor Signaling, Unfolded Protein Response |
| PARP1 | -0.43 | 5.15E-02 | 5.15E-02 | Apoptosis, Disease Association, Oxidative Stress |
| SYT13 | -1.2 | 5.19E-02 | 5.19E-02 | Neural Connectivity, Vesicle Trafficking |
| PIK3CB | 0.509 | 5.20E-02 | 5.20E-02 | Angiogenesis, Apoptosis, Carbohydrate Metabolism, Growth Factor Signaling, Transmitter Release, Transmitter Response and Reuptake, Trophic Factors |
| PRNP | -0.993 | 5.20E-02 | 5.20E-02 | Disease Association, Oxidative Stress |
| MYD88 | 0.399 | 5.35E-02 | 5.35E-02 | Disease Association, Neural Connectivity |
| CXXC1 | 0.329 | 5.37E-02 | 5.37E-02 | Chromatin Modification, Unfolded Protein Response |
| GLS | 0.885 | 5.41E-02 | 5.41E-02 | Carbohydrate Metabolism, Disease Association, Transmitter Release, Vesicle Trafficking |
| PLCL2 | -0.579 | 5.53E-02 | 5.53E-02 | Activated Microglia, Vesicle Trafficking |
| POLR2L | -1.07 | 5.62E-02 | 5.62E-02 | Disease Association, Transcription and Splicing |
| PPP3CB | -0.268 | 5.64E-02 | 5.64E-02 | Disease Association, Growth Factor Signaling, Transmitter Release, Transmitter Response and Reuptake, Vesicle Trafficking |

**Supplementary Table S7. Nanostring differential gene expression AAb vs. ND.** Top differentially expressed genes) for AAb vs ND.

| Gene | Log2 fold change | P-value | Unadj.  p.value | Gene sets |
| --- | --- | --- | --- | --- |
| SQSTM1 | 0.607 | 6.34E-04 | 6.34E-04 | Autophagy, Disease Association |
| TLR2 | 1.46 | 2.34E-03 | 2.34E-03 | Activated Microglia, Disease Association |
| STAT3 | -0.68 | 2.80E-03 | 2.80E-03 | Disease Association, Growth Factor Signaling |
| IL10RA | 1.14 | 3.07E-03 | 3.07E-03 | Cytokines, Disease Association |
| HLA | 1.74 | 4.66E-03 | 4.66E-03 | Disease Association, Tissue Integrity |
| PPP2CA | -0.353 | 9.73E-03 | 9.73E-03 | Carbohydrate Metabolism, Disease Association, Transmitter Release, Transmitter Response and Reuptake |
| ITGAM | 2.61 | 1.22E-02 | 1.22E-02 | Disease Association, Tissue Integrity |
| SMN1 | -0.934 | 1.34E-02 | 1.34E-02 | Disease Association, Transcription and Splicing |
| ATCAY | 0.991 | 1.47E-02 | 1.47E-02 | Axon and Dendrite Structure, Neural Connectivity |
| ITGA7 | 1.06 | 1.47E-02 | 1.47E-02 | Tissue Integrity |
| CYBB | 1.46 | 1.65E-02 | 1.65E-02 | Activated Microglia, Disease Association, Oxidative Stress |
| EGR1 | 1.77 | 1.65E-02 | 1.65E-02 | Disease Association, Growth Factor Signaling |
| TNFRSF1A | -1.55 | 2.07E-02 | 2.07E-02 | Apoptosis, Cytokines, Disease Association, Growth Factor Signaling |
| TGFB1 | 0.693 | 2.82E-02 | 2.82E-02 | Activated Microglia, Axon and Dendrite Structure, Chromatin Modification, Cytokines, Growth Factor Signaling, Myelination |
| GAA | 0.354 | 2.84E-02 | 2.84E-02 | Activated Microglia, Autophagy |
| CD68 | 1.39 | 2.85E-02 | 2.85E-02 | Activated Microglia, Autophagy, Disease Association |
| ATP6V1D | -0.403 | 2.98E-02 | 2.98E-02 | Disease Association, Transmitter Synthesis and Storage |
| LPAR1 | 0.989 | 3.00E-02 | 3.00E-02 | Axon and Dendrite Structure, Myelination, Neural Connectivity, Transmitter Response and Reuptake |
| GRN | 0.471 | 3.57E-02 | 3.57E-02 | Activated Microglia, Disease Association |
| MAPK9 | -0.49 | 3.62E-02 | 3.62E-02 | Apoptosis, Growth Factor Signaling, Transmitter Release, Transmitter Response and Reuptake, Trophic Factors, Unfolded Protein Response |
| MYRF | 0.584 | 3.85E-02 | 3.85E-02 | Myelination |
| CDK7 | -1.09 | 4.00E-02 | 4.00E-02 | Disease Association, Transcription and Splicing |
| TPM1 | -1.61 | 4.07E-02 | 4.07E-02 | Neuronal Cytoskeleton, Oxidative Stress |
| TRIM37 | -0.401 | 4.43E-02 | 4.43E-02 | Chromatin Modification, Unfolded Protein Response |
| NLGN4X | 1.67 | 4.85E-02 | 4.85E-02 | Axon and Dendrite Structure, Neural Connectivity, Tissue Integrity, Vesicle Trafficking |
| SNRPA | -0.392 | 4.97E-02 | 4.97E-02 | Transcription and Splicing |

**LEGENDS TO SUPPLEMENTARY VIDEOS**

**Supplementary Video 1. TH axons innervating a pancreatic arteriole.** A T1D pancreas sample was cleared by PACT and stained using TH (green) to delineate sympathetic axons and varicosities and SMA (red) to identify smooth muscle cells around an intralobular arteriole. The video shows a confocal image z-stack imaged using a 63x oil objective on a Zeiss LSM 710 (92 slices imaged at 0.37 µm each, total 33.8 µm). The TH varicosities are observed in close proximity to SMA cells. Scale bar 20 µm.

**Supplementary Video 2. TH axons and islet endocrine cells.** A control pancreas sample was cleared by PACT and stained using TH (white) to delineate sympathetic axons and varicosities and SCG3 (green) to identify all islet endocrine cells. The video shows a confocal image stack imaged using a 40x air objective on a Zeiss LSM 710 (28 slices imaged at 0.97 µm each, total 26.2 µm). The TH axons and varicosities are observed in close proximity to endocrine cells. Scale bar 10 µm.

**Supplementary Video 3. Islet TH axons and varicosities, α-cells, and arteriole.** A control pancreas sample was cleared by PACT and stained using TH (white) to delineate sympathetic axons and varicosities glucagon (GCG, green) to identify α-cells, and SMA (red) to delineate smooth muscle cells around an islet arteriole. The video shows a cropped region from a confocal image stack imaged using a 20x air objective on a Zeiss LSM 710 (31 slices imaged at 2.29 µm each, total 68.8 µm). The TH axons and varicosities are observed in close proximity to α-cells and distinct from those at the SMA-labeled artery. Scale bar 8 µm.

1 Campbell-Thompson, M. et al. Insulitis and beta-Cell Mass in the Natural History of Type 1 Diabetes. Diabetes 65, 719-731, doi:10.2337/db15-0779 (2016).
