# Supplementary material for: Islet sympathetic innervation and islet neuropathology in patients with type 1 diabetes
Source: Sci Rep. 2021 Mar 22;11:6562. doi: 10.1038/s41598-021-85659-8 (PMC7985489; doi:10.1038/s41598-021-85659-8)
Supplement: Supplementary file 2 — Supplementary Figures. [file 41598_2021_85659_MOESM2_ESM.pptx]

## Slide 1
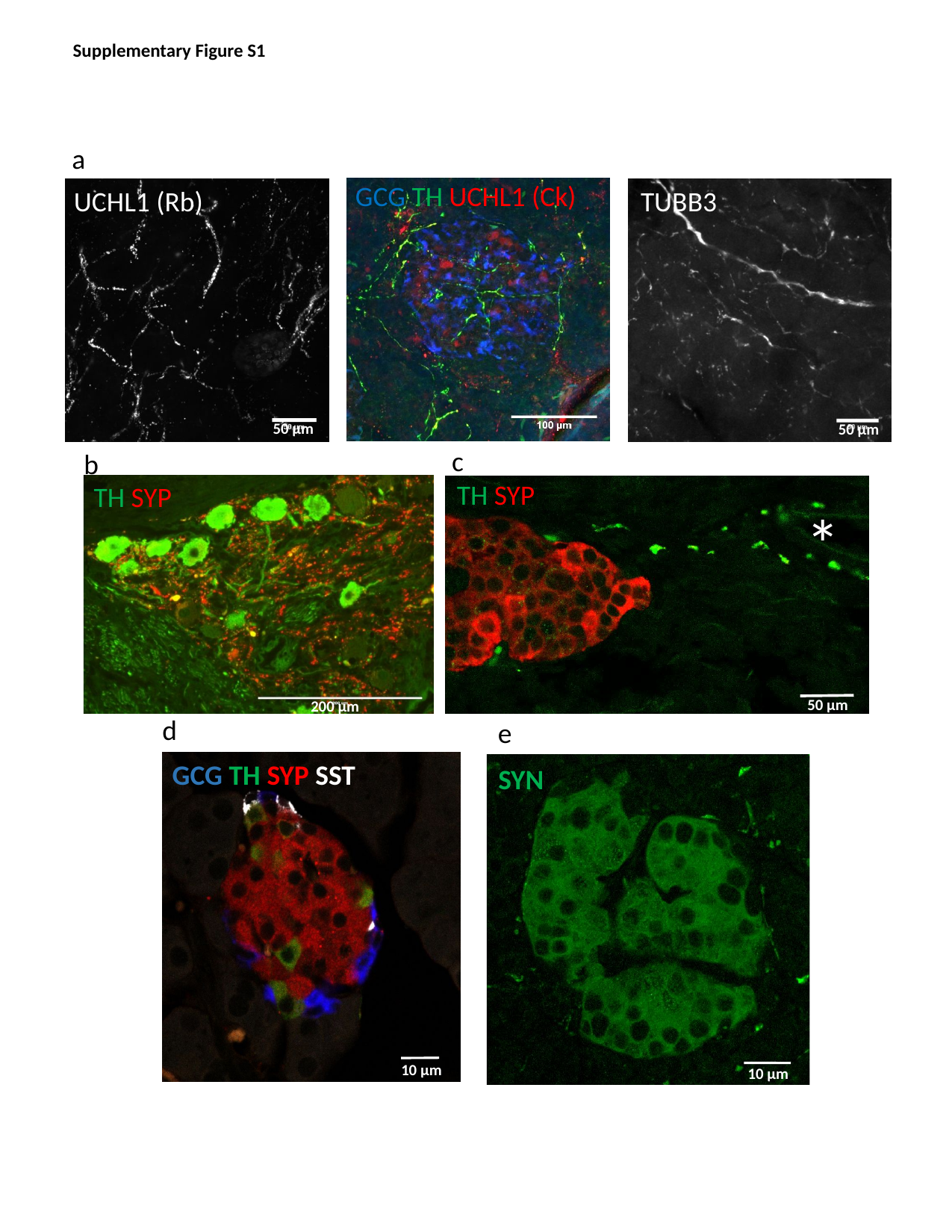

Supplementary Figure S1
a
UCHL1 (Rb)
UCHL1 (Ck)
TUBB3
d
e
GCG TH SYP SST
SYN
GCG TH UCHL1 (Ck)
50 µm
50 µm
c
b
TH SYP
TH SYP
*
50 µm
200 µm
10 µm
10 µm

## Slide 2
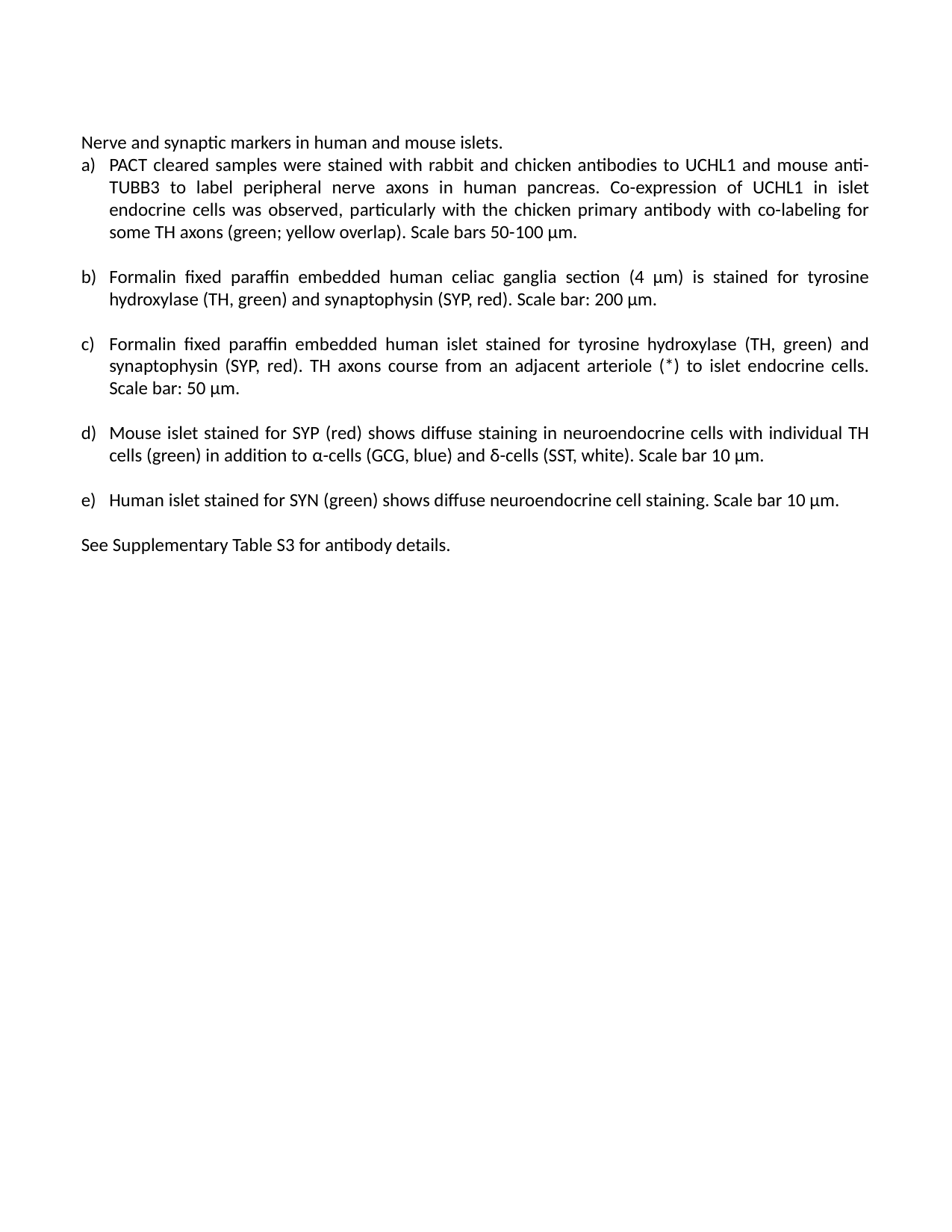

Nerve and synaptic markers in human and mouse islets.
PACT cleared samples were stained with rabbit and chicken antibodies to UCHL1 and mouse anti-TUBB3 to label peripheral nerve axons in human pancreas. Co-expression of UCHL1 in islet endocrine cells was observed, particularly with the chicken primary antibody with co-labeling for some TH axons (green; yellow overlap). Scale bars 50-100 µm.
Formalin fixed paraffin embedded human celiac ganglia section (4 µm) is stained for tyrosine hydroxylase (TH, green) and synaptophysin (SYP, red). Scale bar: 200 µm.
Formalin fixed paraffin embedded human islet stained for tyrosine hydroxylase (TH, green) and synaptophysin (SYP, red). TH axons course from an adjacent arteriole (*) to islet endocrine cells. Scale bar: 50 µm.
Mouse islet stained for SYP (red) shows diffuse staining in neuroendocrine cells with individual TH cells (green) in addition to α-cells (GCG, blue) and δ-cells (SST, white). Scale bar 10 µm.
Human islet stained for SYN (green) shows diffuse neuroendocrine cell staining. Scale bar 10 µm.
See Supplementary Table S3 for antibody details.

## Slide 3
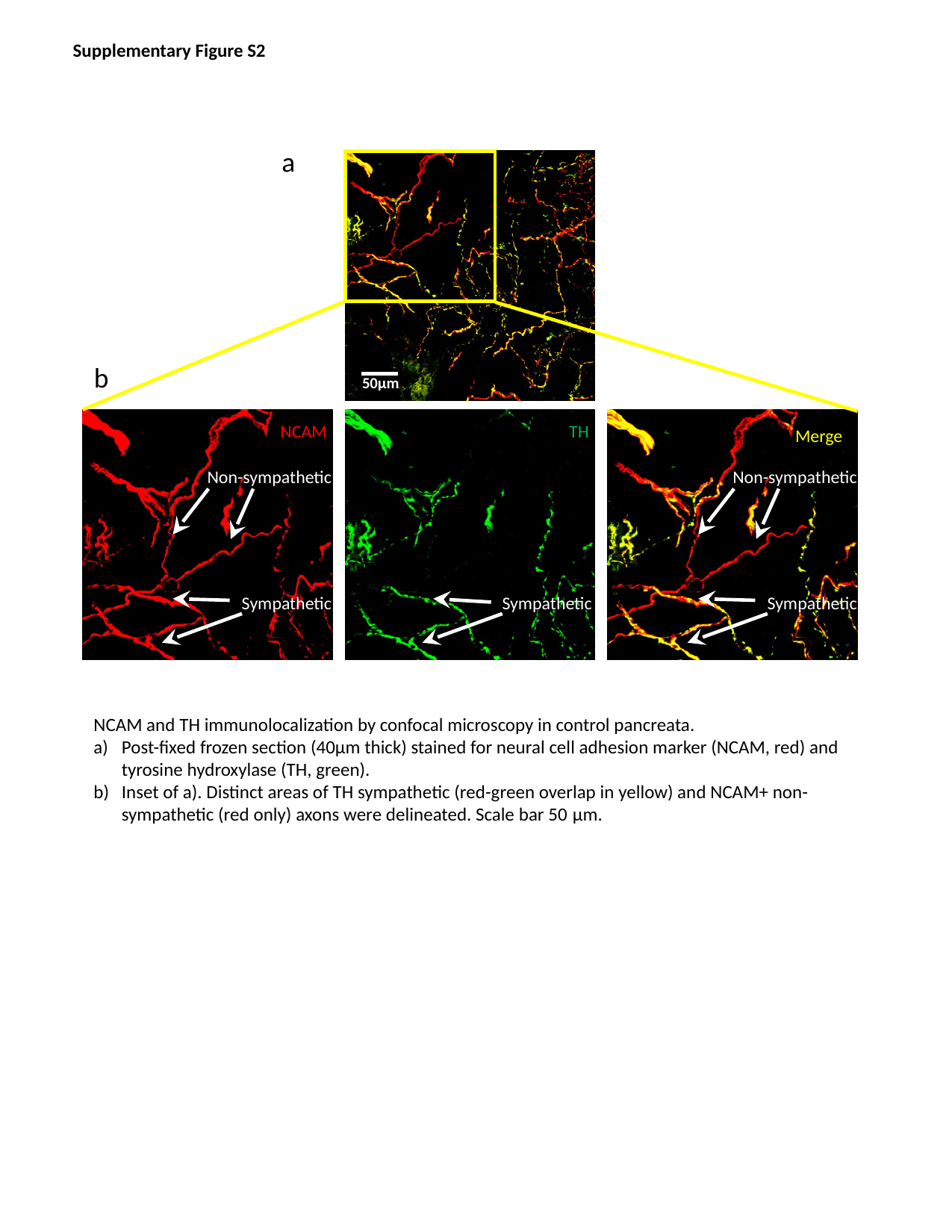

Supplementary Figure S2
a
50µm
NCAM
TH
Merge
Non-sympathetic
Sympathetic
Non-sympathetic
Sympathetic
Sympathetic
b
NCAM and TH immunolocalization by confocal microscopy in control pancreata.
Post-fixed frozen section (40µm thick) stained for neural cell adhesion marker (NCAM, red) and tyrosine hydroxylase (TH, green).
Inset of a). Distinct areas of TH sympathetic (red-green overlap in yellow) and NCAM+ non-sympathetic (red only) axons were delineated. Scale bar 50 µm.

## Slide 4
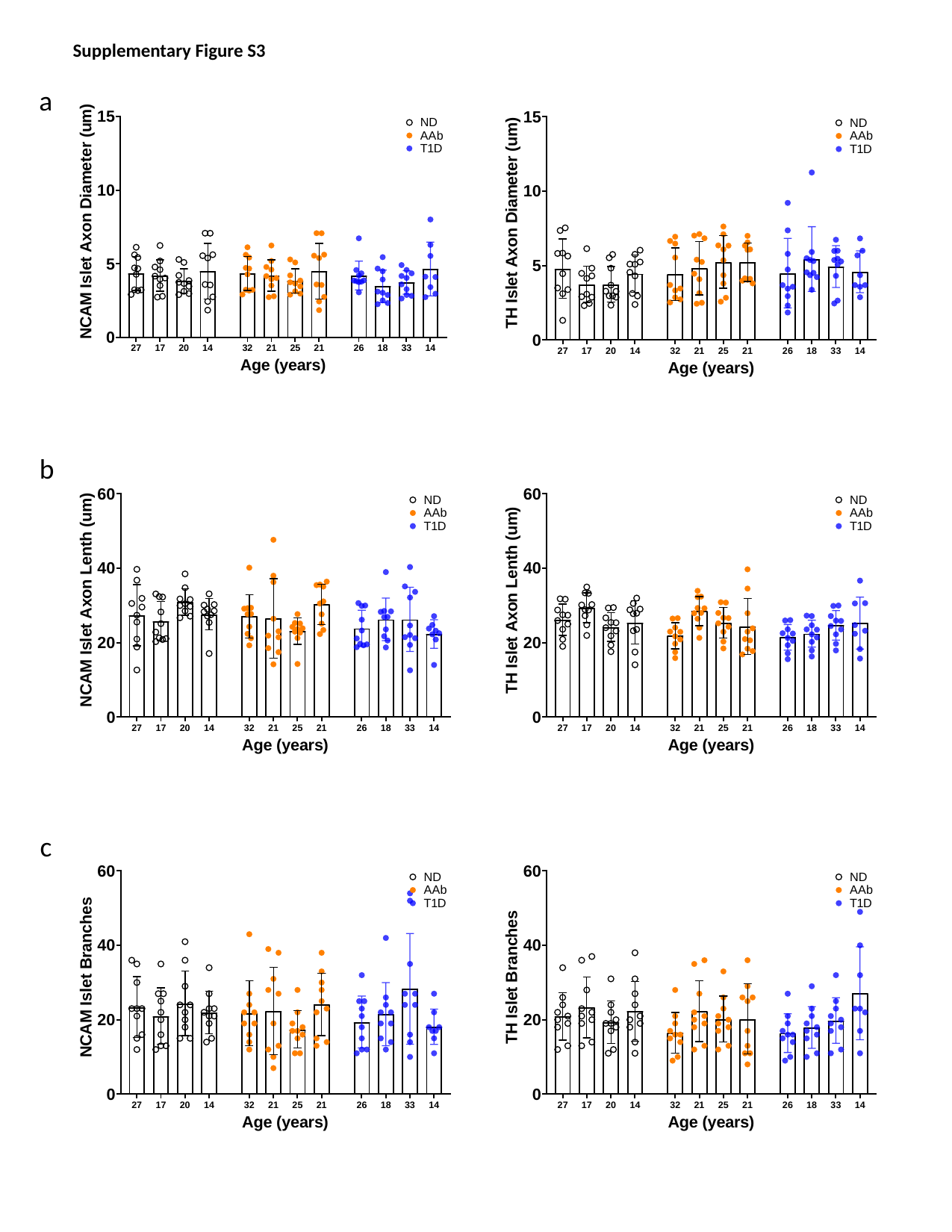

Supplementary Figure S3
a
b
c

## Slide 5
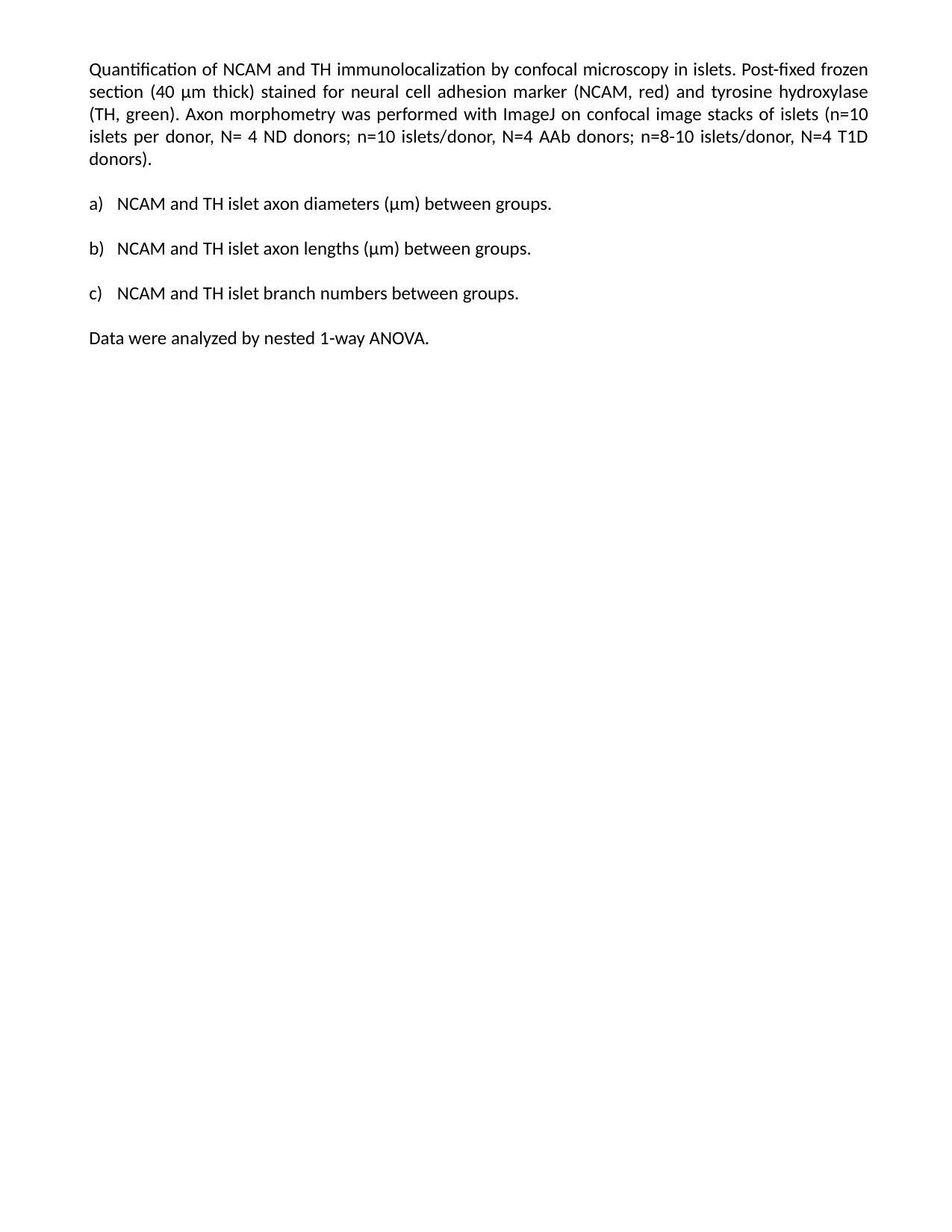

Quantification of NCAM and TH immunolocalization by confocal microscopy in islets. Post-fixed frozen section (40 µm thick) stained for neural cell adhesion marker (NCAM, red) and tyrosine hydroxylase (TH, green). Axon morphometry was performed with ImageJ on confocal image stacks of islets (n=10 islets per donor, N= 4 ND donors; n=10 islets/donor, N=4 AAb donors; n=8-10 islets/donor, N=4 T1D donors).
NCAM and TH islet axon diameters (µm) between groups.
NCAM and TH islet axon lengths (µm) between groups.
NCAM and TH islet branch numbers between groups.
Data were analyzed by nested 1-way ANOVA.

## Slide 6
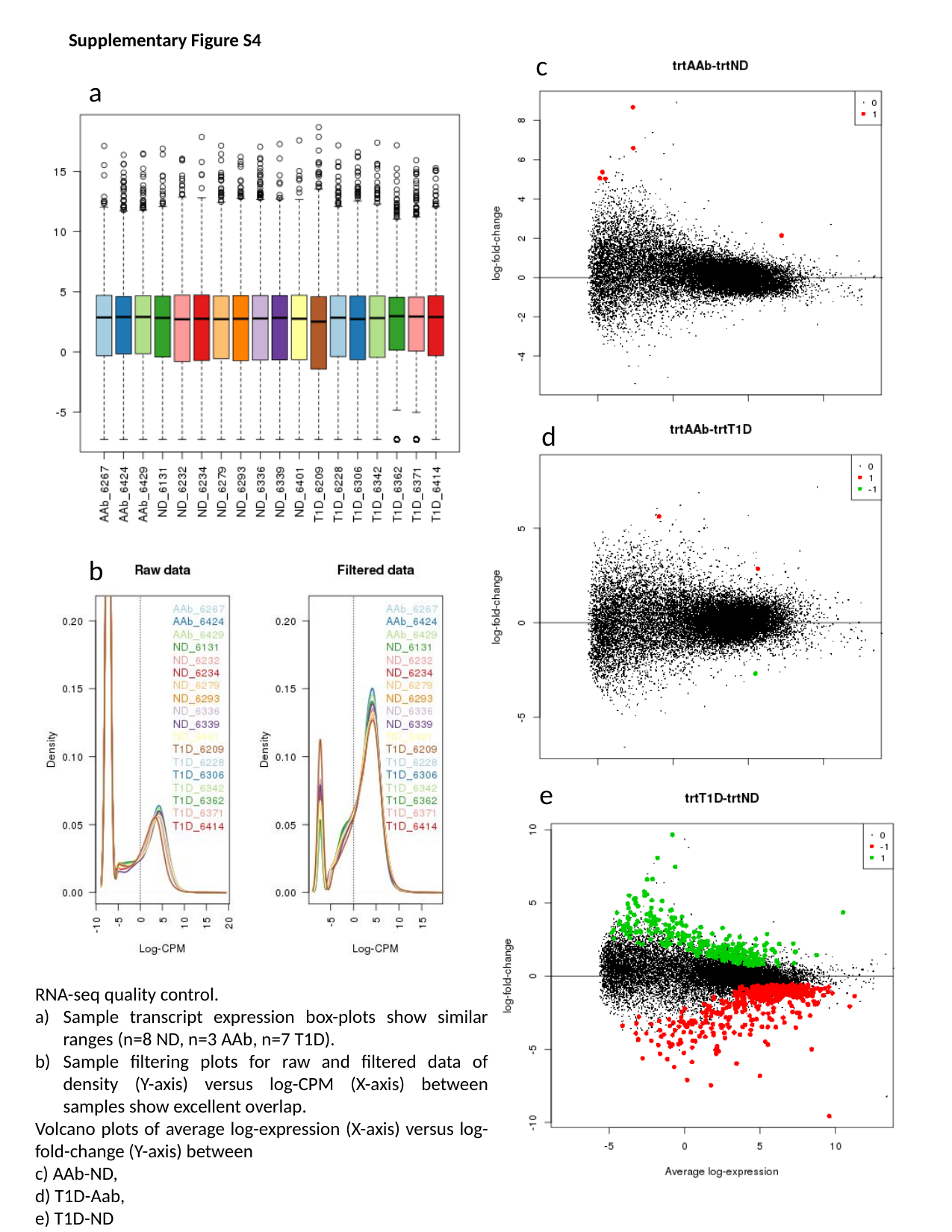

Supplementary Figure S4
c
a
d
b
e
RNA-seq quality control.
Sample transcript expression box-plots show similar ranges (n=8 ND, n=3 AAb, n=7 T1D).
Sample filtering plots for raw and filtered data of density (Y-axis) versus log-CPM (X-axis) between samples show excellent overlap.
Volcano plots of average log-expression (X-axis) versus log-fold-change (Y-axis) between
c) AAb-ND,
d) T1D-Aab,
e) T1D-ND

## Slide 7
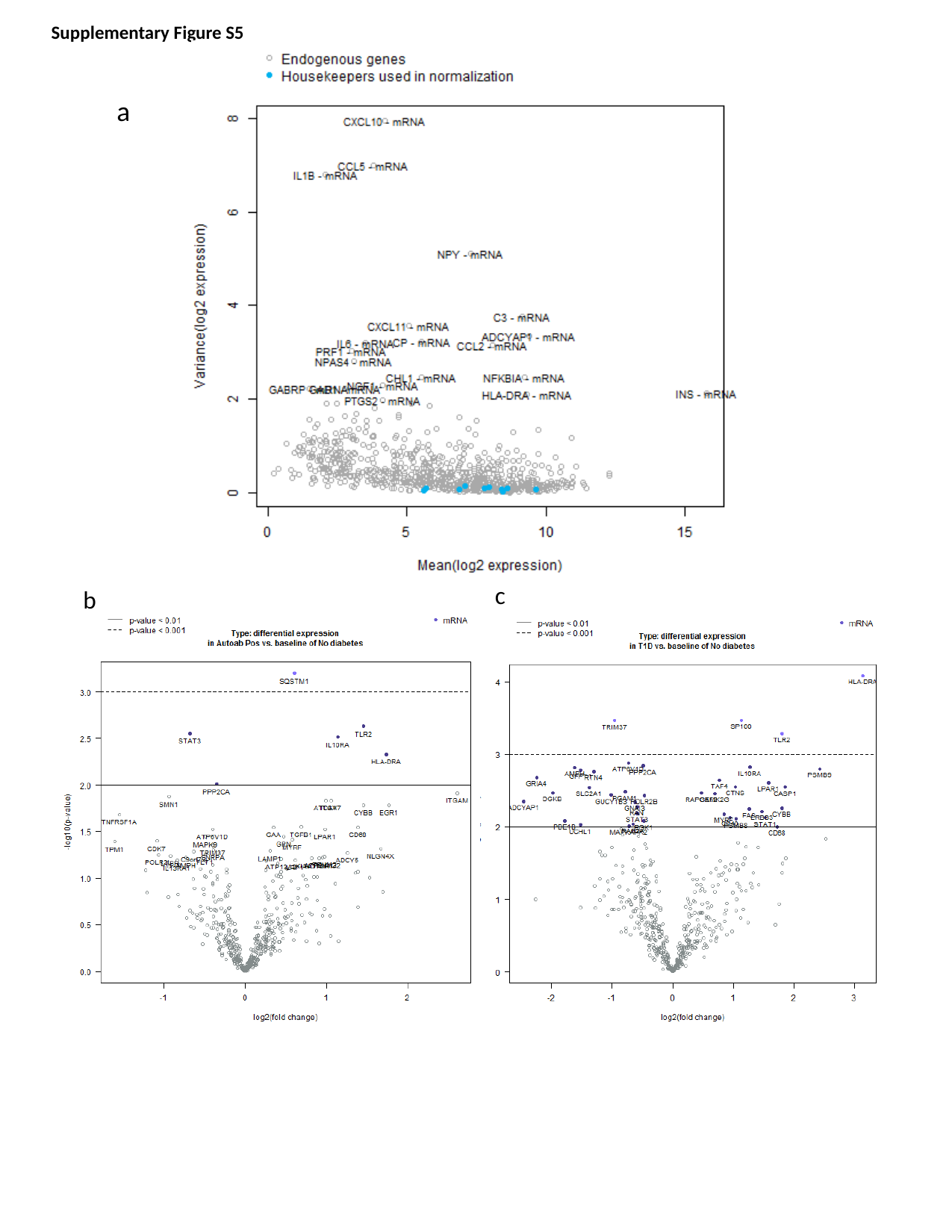

Supplementary Figure S5
a
c
b

## Slide 8
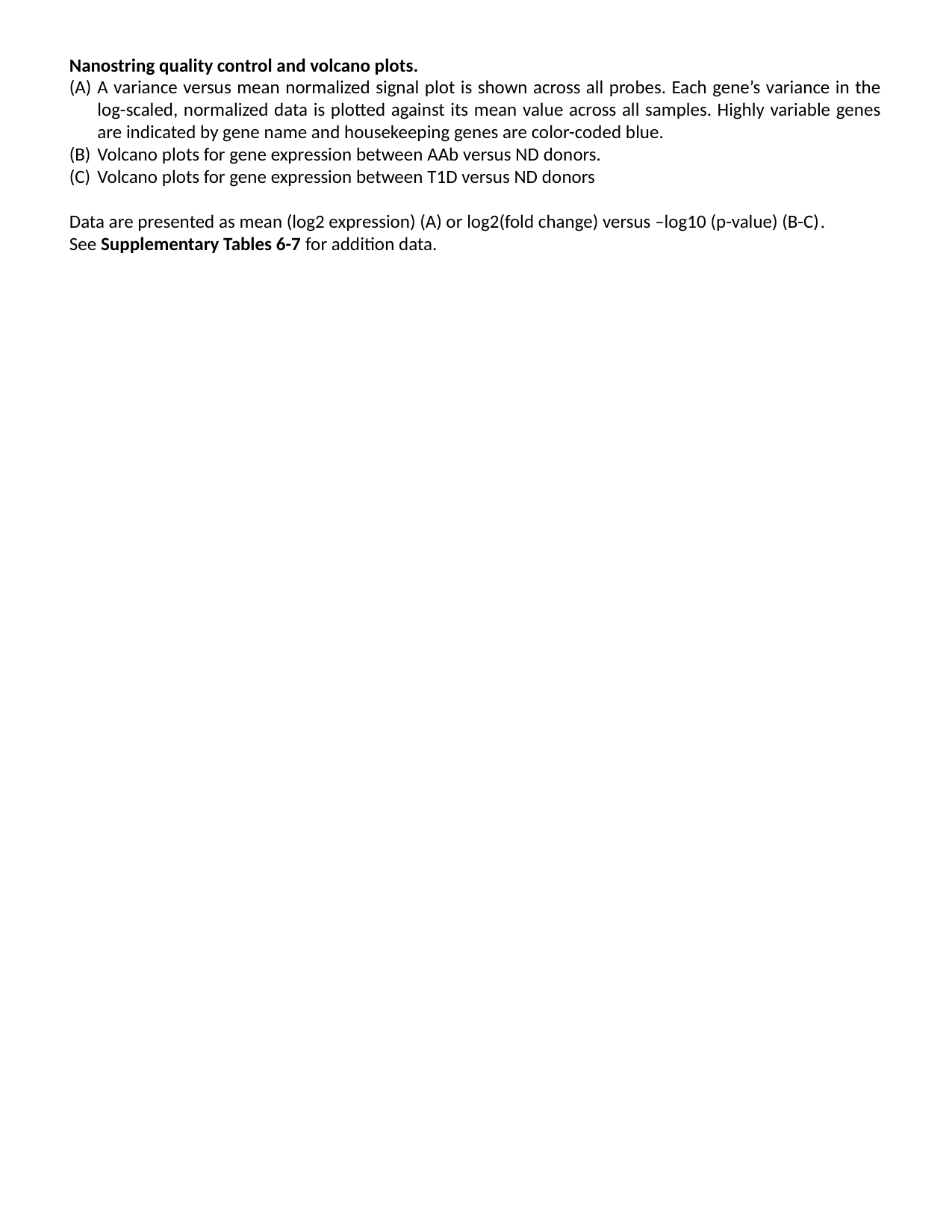

Nanostring quality control and volcano plots.
A variance versus mean normalized signal plot is shown across all probes. Each gene’s variance in the log-scaled, normalized data is plotted against its mean value across all samples. Highly variable genes are indicated by gene name and housekeeping genes are color-coded blue.
Volcano plots for gene expression between AAb versus ND donors.
Volcano plots for gene expression between T1D versus ND donors
Data are presented as mean (log2 expression) (A) or log2(fold change) versus –log10 (p-value) (B-C).
See Supplementary Tables 6-7 for addition data.
